# Integrated hepatic transcriptomics and metabolomics identify Pck1 as a key factor in the broad dysregulation induced by vehicle pollutants

**DOI:** 10.1186/s12989-024-00605-6

**Published:** 2024-12-30

**Authors:** Gajalakshmi Ramanathan, Yuqi Zhao, Rajat Gupta, Siri Langmo, May Bhetraratana, Fen Yin, Will Driscoll, Jerry Ricks, Allen Louie, James A. Stewart, Timothy R. Gould, Timothy V. Larson, Joel Kaufman, Michael E. Rosenfeld, Xia Yang, Jesus A. Araujo

**Affiliations:** 1 Division of Cardiology, David Geffen School of Medicine, University of California-Los Angeles, 10833 Le Conte Avenue, CHS 43-264, P.O. Box 951679, Los Angeles, CA 90095 USA; 2 Department of Integrative Biology and Physiology, University of California-Los Angeles, Los Angeles, CA USA; 3 Environmental and Molecular Toxicology Interdepartmental Program, University of California-Los Angeles, Los Angeles, CA USA; 4 Department of Environmental Health Sciences, Fielding School of Public Health, University of California-Los Angeles, Los Angeles, CA USA; 5 Department of Pathology, University of Washington, Seattle, WA USA; 6 Department of Environmental and Occupational Health Sciences, University of Washington, Seattle, WA USA; 7 Department of Civil and Environmental Engineering, University of Washington, Seattle, WA USA; 8 Molecular Biology Institute, University of California-Los Angeles, Los Angeles, CA USA

**Keywords:** Diesel exhaust, Air pollution, Liver, Transcriptomics, Metabolomics, Mitochondrial dysfunction, Glycogenolysis, Gluconeogenesis, Pck1

## Abstract

**Background:**

Exposure to air pollution is associated with worldwide morbidity and mortality. Diesel exhaust (DE) emissions are important contributors which induce vascular inflammation and metabolic disturbances by unknown mechanisms. We aimed to determine molecular pathways activated by DE in the liver that could be responsible for its cardiometabolic toxicity.

**Methods:**

Apolipoprotein E knockout (ApoE KO) mice were exposed to DE or filtered air (FA) for two weeks, or DE for two weeks followed by FA for 1 week. Expression microarrays and global metabolomics assessment were performed in the liver. An integrated transcriptomic and metabolomic analytical strategy was employed to dissect critical pathways and identify candidate genes that could dissect DE-induced pathogenesis. HepG2 cells were treated with an organic extract of DE particles (DEP) vs. vehicle control to test candidate genes.

**Results:**

DE exposure for 2 weeks dysregulated 658 liver genes overrepresented in whole cell metabolic pathways, especially including lipid and carbohydrate metabolism, and the respiratory electron transport pathway. DE exposure significantly dysregulated 118 metabolites, resulting in increased levels of triglycerides and fatty acids due to mitochondrial dysfunction as well as increased levels of glucose and oligosaccharides. Consistently, DEP treatment of HepG2 cells led to increased gluconeogenesis and glycogenolysis indicating the ability of the in-vitro approach to model effects induced by DE in vivo. As an example, while gene network analysis of DE livers identified phosphoenolpyruvate carboxykinase 1 *(Pck1)* as a key driver gene of DE response, DEP treatment of HepG2 cells resulted in increased mRNA expression of *Pck1* and glucose production, the latter replicated in mouse primary hepatocytes. Importantly, *Pck1* inhibitor mercaptopicolinic acid suppressed DE-induced glucose production in HepG2 cells indicating that DE-induced elevation of hepatic glucose was due in part to upregulation of Pck1 and increased gluconeogenesis.

**Conclusions:**

Short-term exposure to DE induced widespread alterations in metabolic pathways in the liver of ApoE KO mice, especially involving carbohydrate and lipid metabolism, together with mitochondrial dysfunction. *Pck1* was identified as a key driver gene regulating increased glucose production by activation of the gluconeogenesis pathway.

**Supplementary Information:**

The online version contains supplementary material available at 10.1186/s12989-024-00605-6.

## Introduction

Air pollution exposure has long been linked to a broad spectrum of health effects, resulting in cancer, cardiovascular (CV) and cerebrovascular diseases [1, 2]. Recent studies in both humans and animals suggest that air pollution is also an important risk factor for type 2 diabetes mellitus (T2DM) [3], obesity, and Non-alcoholic Fatty Liver Disease (NAFLD) [4, 5]. It is possible that the metabolic effects of air pollution are at the intersection of CV, endocrinological and gastrointestinal diseases [6]. Unfortunately, the mechanisms by which air pollution induces diabetes, NAFLD, and how they connect with CV diseases are not well understood.

Growing evidence indicates a complex dynamics between several pathways leading to metabolic dysregulation, which include the induction of systemic oxidative stress and inflammation [7], endoplasmic reticulum stress [8] and mitochondrial dysfunction [9, 10]. Experimental studies suggest the involvement of tissues participating in the pathogenesis of T2DM and NAFLD such as liver, adipose tissue, and the immune system [6]. We have previously shown that apolipoprotein E (ApoE) knockout (KO) mice, exposed to ambient ultrafine particles and/or diesel exhaust (DE), exhibit systemic acute pro-oxidant and pro-inflammatory effects as early as 2 weeks after exposures, evidenced by increased plasma levels of 8-isoprostanes, 12-hydroxyeicosatetraenoic (12-HETE) acid and 13-hydroxyoctadecadienoic (13-HODE) acid, development of dysfunctional high density lipoprotein cholesterol (HDL-c), characterized by loss of its anti-oxidant/anti-inflammatory properties, and activation of the 5-lipoxygenase (5-LO) pathway in the liver [7, 11]. When exposures continue for 16 weeks, mice develop hypertriglyceridemia and liver steatosis due to decreased lipid catabolism [12]. Importantly, young healthy individuals develop similar oxidative effects in plasma lipoproteins when exposed to high levels of ambient pollutants for 6 to 8 weeks when traveling from less polluted Los Angeles to more polluted Beijing [13]. It is unknown, however, whether effects in the circulating blood and vasculature could be related to effects induced in the liver.

Collectively, the liver is a major metabolic organ heavily involved in air pollutant-induced disorders. It is plausible that effects induced in the liver could lead to metabolic derangements responsible for the development of endocrinological diseases (e.g. diabetes), gastrointestinal disorders (e.g. NAFLD) and CV diseases (e.g. atherosclerosis). Thus, low density lipoprotein receptor (Ldl-R) knockout mice exposed to ultrafine particles (UFP) develop an atherogenic plasma lipid profile [14], which could be related to metabolic effects in the liver. Other studies have shown that exposures to PM_2.5_ induce profound effects on the liver as well, including oxidative stress, endoplasmic reticulum (ER) stress, and inflammatory responses that eventually lead to a metabolic phenotype that includes hepatic steatosis, inflammation, fibrosis, and insulin resistance [4, 5]. Therefore, understanding molecular pathways activated in the liver early after the exposures to PM is of paramount importance.

The current study aimed at dissecting gene and metabolic pathways activated in the liver early, 2 weeks after exposing ApoE KO mice to diesel exhaust, an important source of motor vehicle air pollutants, with the use of an integrated transcriptomic and metabolomics approach. We also employed gene network analysis to identify candidate genes in specific biological networks, potentially responsible for liver metabolic effects, followed by in-vitro experimentation for causal validation of those candidate genes.

## Materials and methods

### Study design, DE generation and animal exposures

Male ApoE^−/−^ mice on the C57BL/6 background at approximately 8 weeks of age were subjected to exposure to DE, filtered air (FA), or DE followed by FA in 2 separate experimental protocols as summarized in Table 1, which enabled inter-experimental validation of data obtained in each individual protocol. The exposure protocol has been previously described [7]. Briefly, male mice were bred and housed at the University of Washington South Lake Union animal facility. Mice were allowed access to water and standard rodent chow. Protocol 1 consisted of mice assigned to three experimental groups (*n* = 12–13/group) that were exposed to: (A) DE for 2 weeks (*n* = 12), (B) FA for 2 weeks as controls (*n* = 13), and (C) DE for 2 weeks followed by FA for 1 additional week (DE + FA, *n* = 13). In Protocol 2, mice were assigned to two experimental groups (*n* = 10–12/group), exposed to almost identical conditions as groups A (*n* = 10) and B (*n* = 12) of Protocol 1. Exposures were carried out 6 h/day, 5 days/week and exposure conditions were almost identical for both protocols. DE particulate, as fine particulate exposure mass concentrations, were measured in real-time. The mass concentration was monitored during exposures with a TEOM analyzer (Rupprecht & Patashnick Model 1400a) and an integrating light-scattering nephelometer (Radiance Research Model 903). The average PM_2.5_ concentrations were 258 µg/m^3^ (SD 39) and 253 µg/m^3^ (SD 5) in Protocols 1 and 2, respectively. More detailed aerosol characteristics (particle number, mass median aerodynamic diameter, mass distribution, particle bound polycyclic aromatic hydrocarbons-PAHs, carbon fractionation of particulate) were conducted on spot measurements as reported [7], yielding a mass mean aerodynamic diameter of 77 nm (GSD 7.4 nm) using a MOUDI impactor, particle number concentration of 145,000 particles/cm^3^ (SD 5400), particle bound PAHs of 21 ng/mg PM_2.5_ (SD 2), and ratio of organic to elemental carbon in the particles: 0.1 (SD 0.02); since exposure generation methods were consistent, they are expected to be representative of the entire period. The concentrations of oxides of nitrogen for both protocols were at 1220 ppb NO (SD 160) and 45 ppb NO2 (SD 17), with average values of 1302 ppb NO and 46 ppb NO2 for Protocol 1, and 1040 ppb NO and 28 ppb NO2 for Protocol 2, measured using a Thermo Scientific Model 42 C analyzer. The concentration of carbon monoxide was 1.3 ppm (SD 0.1), with average values of 1.38 ppb for Protocol 1 and 1.30 ppb for Protocol 2, measured using a Langan analyzer, Model T15n. Animal procedures were approved by the Animal Care and Use Committees of the University of Washington and UCLA. Protocol 1 was used for transcriptional profiling and Protocol 2 was used for metabolic signatures, as depicted in Table 1.

**Table 1:** Experimental protocols

#	Animal strain	Groups	Time	Diet	Analysis
1	Male ApoE-/-	DE	2 wk	Chow	Transcriptomics, liver, *n* = 8/group
		FA	2 wk		
		DE + FA	2 wk + 1 wk		
2	Male ApoE-/-	DE	2 wk	Chow	Metabolomics, liver, *n* = 5/group
		FA	2 wk		

Transcriptomic analysis was conducted in ApoE null mice exposed to diesel exhaust (DE) or filtered air (FA) for 2 weeks (wk), or DE for 2 weeks followed by one week of FA (DE + FA) in Protocol 1. Livers were harvested for RNA extraction and Illumina microarrays, *n* = 8 per group. Metabolomics was performed on livers from ApoE null mice exposed to DE or FA for two weeks in Protocol 2. *n* = 5 per group

### RNA preparation and expression microarray analyses

Liver RNA was isolated using the RNeasy kit (Qiagen, Valencia, CA) and analyzed using the BioTek instrument for nucleic acid quantification and integrity. Biotin-labeled cRNA was synthesized by the Total prep RNA amplification kit from Ambion (Austin, TX). cRNA was quantified and normalized to 77 ng/µl, and then 850 ng was hybridized to Beadchips (MouseRef-8 v2.0 BeadChip, Illumina, San Diego, CA). The hybridized Beadchips were scanned by an Illumina BeadScan confocal scanner and analyzed by Illumina’s BeadStudio software, version 1.5.1.3. cRNA synthesis, hybridization and scanning were performed at the UCLA Illumina microarray core facility. The microarray data (available at Mendeley Data,  https://data.mendeley.com/datasets/6c9ctnn2dg/1) was normalized by the rank invariant method and analyzed using Illumina’s GenomeStudio Gene Expression Module v1.0. After normalization of the transcriptomics dataset, differential expression was performed using the Illumina Custom algorithm, by assigning FA as the reference group. False discovery rate (FDR) was computed by applying multiple testing correction using the Benjamini and Hochberg correction at 5% cutoff. Differential *p*-value ≤ 0.05 after adjusting for FDR was considered statistically significant. The heatmap of differentially expressed genes (DEGs) was prepared using R. The DE group was compared to the FA group to derive DEGs for DE exposure. The DE + FA group was also compared to the FA group to derive DEGs for DE + FA. Gene Set Enrichment Analysis (GSEA) was performed to detect significantly enriched gene sets and pathways curated in the Molecular Signature Database (MsigDB), and to derive FDR [15, 16]. FDR < 0.05 was used to determine significance.

### Network modeling and identification of key drivers (KD) of DE

We constructed liver-specific Bayesian networks (BNs) based on genetic and transcriptomic data from multiple large-scale mouse studies [17]. We used the DEGs of DE and DE + FA groups separately as seeds to extract the top most connected subnetworks as described previously [18]. The network components including nodes and edges as well as topological structures were visualized using Cytoscape [19]. A Key Driver Analysis (KDA) [20, 21] was used to identify potential key regulators for the DE or DE + FA gene signatures based on the topology of BNs. Briefly, the neighboring subnetwork of each gene in the network was first extracted and then compared with the DE or DE + FA gene signatures to assess the enrichment of genes in the latter using Fisher’s exact test. Network genes that reach Bonferroni-adjusted *p* < 0.05 were reported as KDs.

### Preparation of DEP extract

Diesel exhaust particles (DEP) were obtained as a generous gift from Dr. David Diaz-Sanchez while at UCLA, originally generated at the National Institute of Environmental Studies (Tsukuba, Ibaraki, Japan). DEP were collected from four-cylinder Isuzu diesel engine (Isuzu, Hokkaido, Japan) and the chemical composition has been previously characterized, including PAH and quinone content [22–24]. A methanol extract of the DEP was prepared as described before [25]. Briefly, DEP was resuspended in methanol, sonicated on ice, and centrifuged. The supernatant was dried under nitrogen gas, the extract was dissolved in DMSO and the dark at − 80 °C until use. DEP components are shown in Supplementary Tables 1, and the chemical composition of this extract, including the presence of the redox cycling organic substances such as polycyclic aromatic hydrocarbons and quinones are shown in Supplementary Tables 2 and 3.

### Tissue culture and metabolic studies for KD validation in HepG2 cells

HepG2 cells were obtained from ATCC and cultured in DMEM low glucose (1 g/l or 5.5 mM) media supplemented with 10% FBS, penicillin (100 units/ml) and streptomycin (100 µg/ml). Cells were passaged every 2–3 days using 0.25% Trypsin-EDTA solution. Cultures were incubated at 37 °C in humidified atmosphere with 5% CO_2_. To investigate glycogenolysis, HepG2 cells were treated with 100 µg/ml DEP methanol extract for 1 h and then stained with Periodic acid Schiff (PAS) reagent kit (Sigma). PAS stain was quantified using ImageJ software. 100 µg/ml DEP extract is a high concentration that could be representative of what cells in the bifurcations within the respiratory tract of a high-risk individual (i.e., someone who lives/works near particle sources) may be exposed to [26], and although nanoparticles could translocate into systemic tissues [27], there is no in-vivo data available indicating what concentrations of particle chemical constituents, hepatocytes could be subjected to, after exposure to DE. To assess gluconeogenesis, we employed a glucose production assay where HepG2 cells were seeded in 24-well plates (2 × 10^5^ cells per well) and treated in serum-free DMEM with 100 µg/ml DEP for 8 h. The medium was then replaced with glucose production buffer consisting of glucose-free DMEM (pH 7.4) without phenol red, supplemented with gluconeogenic substrates, 20 mM sodium lactate and 2 mM sodium pyruvate, as previously described [28]. After 24 h incubation, glucose concentration in media was measured using a colorimetric glucose assay kit (Sigma). The readings were then normalized to the total protein content determined from the whole-cell lysates. To determine whether DEP-induced glucose production was due to increased *Pck1* expression and increased gluconeogenesis, cells were treated with DEP along with 3-mercaptopicolinic acid (3-MPA), an inhibitor for *Pck1* enzymatic activity, at 1 mM for 8 h. Cells were then incubated in a glucose production buffer for an additional 24 h, and glucose and total protein concentrations were measured. To assess cell viability, we employed a thiazolyl blue tetrazolium (MTT) assay where HepG2 cells were seeded in 96-well plates (2 × 10^4^ cells per well). The cells were initially serum starved for 6 h and then treated in serum-free DMEM with 100 µg/ml DEP for 24 h. Following a previously published protocol [25], 20 ul of MTT (1.2 mg/ml in media) was added to each well at the end of the treatments, and the plate was incubated for an additional 2 h, then with 100 ul of DMSO for an additional 10 min, after which absorbance was measured at 560 nm.

### Isolation of primary hepatocytes and validation of glucose production

Primary hepatocytes were isolated from C57BL/6J mice and cultured following a previously published protocol [29] with minor modifications. Briefly, mice were anesthetized using 3% isoflurane, and the liver was perfused with a buffer (PBS with 0.5 M EDTA and 25 mM HEPES) through the Inferior Vena Cava (IVC) at a flow rate of 3 ml/min using a peristaltic pump (Model#: 77122-32, Master Flex, LLC). Once the liver was clear of residual blood, this process was repeated with 10 ml PBS containing 25 L/mL Liberase enzyme (Sigma Aldrich, 5401119001) to initiate liver digestion. Following perfusion, the liver was fully dissected and transferred into a tube with chilled digestion buffer (PBS with 25 mM HEPES). The cells were released from the liver by rupturing the liver sack in a 10 cm plate with the digestion buffer and DMEM low glucose (1 g/l or 5.5 mM) media supplemented with 5% FBS, penicillin (100 units/ml) and streptomycin (100 µg/ml). The hepatocytes were purified by straining the liver, digestion buffer, and cell media mix through a 70-µm cell strainer. The resulting mixture was then spun at 50 × g for 2 min at 4 °C. The supernatant was aspirated and the hepatocyte pellet was gently resuspended in 10 mL cell media. This was repeated a second time. The isolated primary hepatocytes were then seeded in 12-well plates (collagen-coated) (2 × 10^5^ cells per well) and incubated overnight at 37 °C in humidified atmosphere with 5% CO_2_ before they were treated with 25 µg/ml DEP vs. vehicle for 8 h to assess new glucose production, using the same procedure for HepG2 cells as indicated above.

### Biochemical assays

Liver triglyceride content was determined using the triglyceride colorimetric assay kit (Cayman Chemicals, Ann Arbor, MI). The assay was performed using liver homogenates prepared from mice in FA and DE groups in Protocol 1, following manufacturer’s instructions.

### Quantitative real-time PCR

mRNA expression of HepG2 cells treated with 100 ug/ml DEP methanol extract for 8 h was assessed by real-time qPCR. Cytoplasmic RNA was isolated from HepG2 cells using TRIZOL reagent (Invitrogen, Carlsbad, CA) and cDNA was synthesized using cDNA Synthesis kit (Applied Biosystems). Primers were designed (Integrated DNA technologies) to amplify human genes, Pck1, G6Pc, Igfbp1, Foxo1 and β-2-microglobulin. Quantitative PCR (qPCR) was performed using a LightCycler 480 (Roche Molecular Biochemicals), using probes from the Universal Probe Library (Roche Life Sciences) according to manufacturer’s protocols. qPCR primer sequences are listed in Supplementary Table 4. PCR conditions were: 95ºC for 3 min, 40 cycles of 95ºC for 15 s, 60ºC for 30 s and 72ºC for 30 s. Cp values were determined using the second derivative analysis (LightCycler Relative Quantification Software). Samples were then normalized to β-2-microglobulin measured by qPCR for each sample.

### Mitochondrial respiration

Frozen liver tissues were stored in -80 C until prepared for the Seahorse experiments. Frozen tissues were thawed on ice and homogenized in MAS buffer (70 mM sucrose, 220 mM mannitol, 5 mM KH2PO4, 5 mM MgCl2, 1 mM EGTA, 2 mM HEPES pH 7.2) with a bead homogenizer. A BeadBlaster (Benchmark Scientific) was set to homogenize with 2 cycles of 30 s on, 30 s off at 6.5 m/s. The beads were washed with 50 µL MAS to recover the remaining homogenate. Homogenates were centrifuged at 500 x g and the supernatant was collected for testing. Protein concentrations were determined by BCA assay kit (Thermo Fisher). Homogenates were loaded into Seahorse XF96 microplate at 12 µg/well in MAS buffer (20 µL each well) and centrifuged at 2,000×g for 5 min at 4° C. After centrifugation the volume was increased to 150 µL by adding 130 µL MAS containing cytochrome c (10 µg/mL). Substrate injections at port A included final concentrations of 1 mM NADH to determine the respiratory capacity of mitochondrial Complex I or 5 mM succinate with 2 µM rotenone to determine the respiratory capacity of mitochondrial Complex II. The following compounds were injected sequentially to final concentration of 2 µM rotenone with 4 µM antimycin A (Port B); 0.5 mM TMPD with 1 mM ascorbic acid (Port C); and 50 mM azide (Port D). OCR rates were measured using Seahorse XF96 Extracellular Flux Analyzer (Agilent Technologies) and normalized to protein or mitochondrial content quantified by MitoTracker Deep Red (MTDR). For MTDR normalization, 3 µg/well of homogenate was stained with 500 nM MTDR for 10 min followed by two wash steps to remove the dye (Thermo Fisher). MTDR fluorescence was read on a Tecan Spark plate reader (Ex: 633 nm; Em: 678 nm).

### Metabolomics

Liver global metabolomic profiling was performed by Metabolon Inc. (Durham, NC). Frozen liver samples from Protocol 2 were used for this analysis. The protocol for metabolic profiling as detailed by Metabolon Inc. can be found in the Supplemental Methods. Random Forest (RF) is a supervised classification technique reporting on the consensus of a large number of decision trees. Liver samples from FA or DE were classified in order to: (1) assess the capacity of metabolomics to distinguish between the FA (control) and DE groups based on overall metabolic profiles and (2) identify key metabolites important to the classification. Welch’s two-sample t-test was used to identify biochemicals that differed significantly between experimental groups after an estimate of the false discovery rate (*q*-value < 0.12) was calculated to take into account the multiple comparisons. *p*-value of ≤ 0.05 was considered statistically significant. Fold enrichment of metabolites in KEGG pathways was defined as:

Enrichment = $$\:\frac{k/K}{n/N}$$ where;

k- # of significant metabolites in pathway.

K- # of detected metabolites in pathway.

n- total # of significant metabolites.

N- total # of detected metabolites.

### Statistics

For the biochemical and qPCR experiments, data are expressed as mean ± SEM. Unpaired two-tailed Student’s t-test or Mann-Whitney U test were used for comparisons between the control and DE groups. One-way ANOVA followed by post-hoc Tukey’s test was used for comparisons among 3 or more groups or conditions. Differences were considered statistically significant at the *p*-value of ≤ 0.05. Statistical analyses for the transcriptomic and metabolomic data are described under their respective experimental methodology.

## Results

We aimed to identify transcriptional and biochemical changes taking place in the liver following exposure to DE. Groups of male ApoE KO mice fed a chow diet were exposed to DE or FA for two weeks and their livers harvested for transcriptional (Protocol 1) and biochemical (Protocol 2) analysis.

### DE-induced gene dysregulation in the liver

**Fig. 1:**
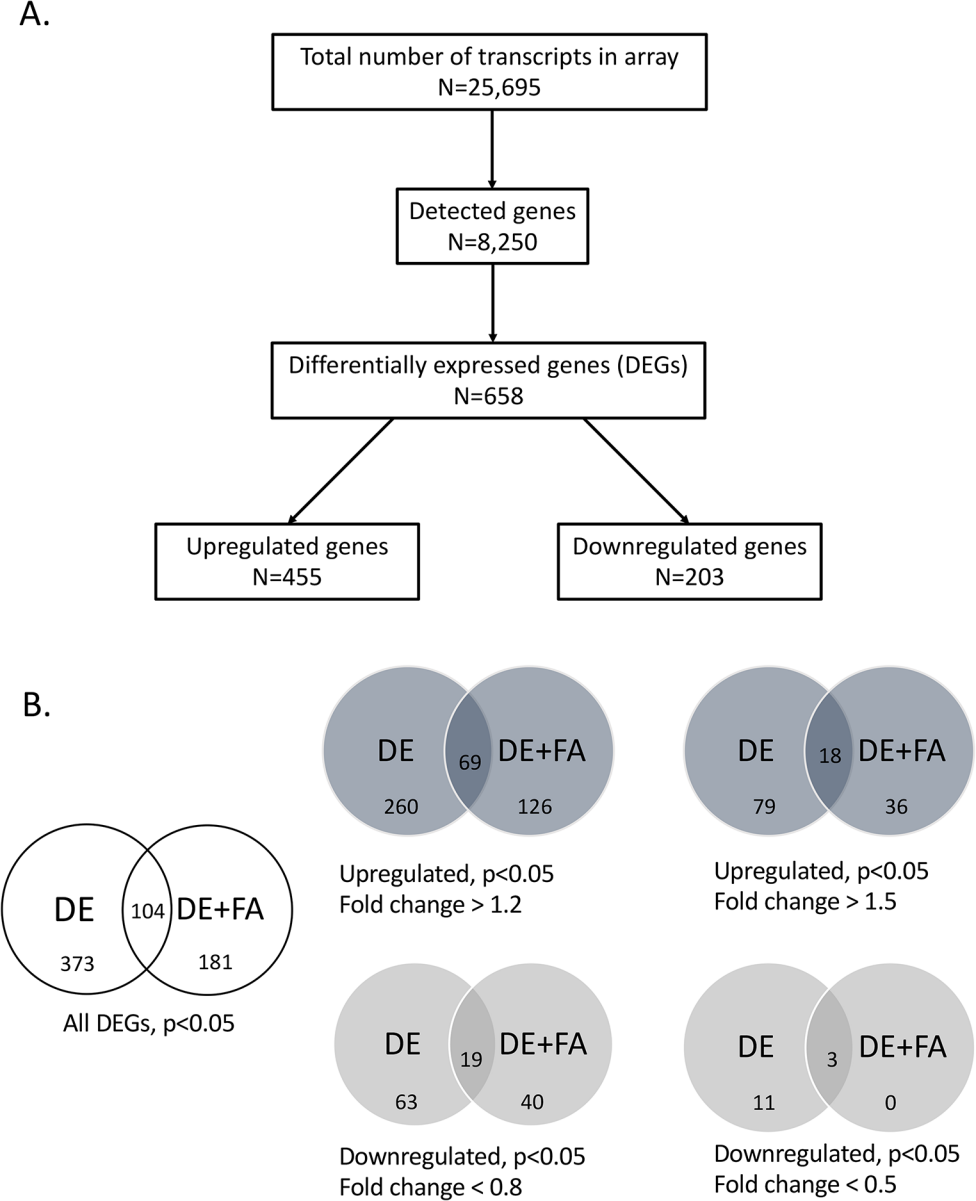
Summary of transcriptomic data. (**A**) Flowgram of numbers of genes in the microarray dataset. (**B**) Venn diagrams of differentially expressed genes (DEGs) compared to the filtered air group (*p* < 0.05). The top panel shows the upregulated DEGs from the DE and DE + FA groups. The bottom panel shows the downregulated DEGs from the DE and DE + FA groups. Values displayed in the circle intersections indicate the number of genes commonly altered by both conditions, *n*= 8 mice/group

**Fig. 2:**
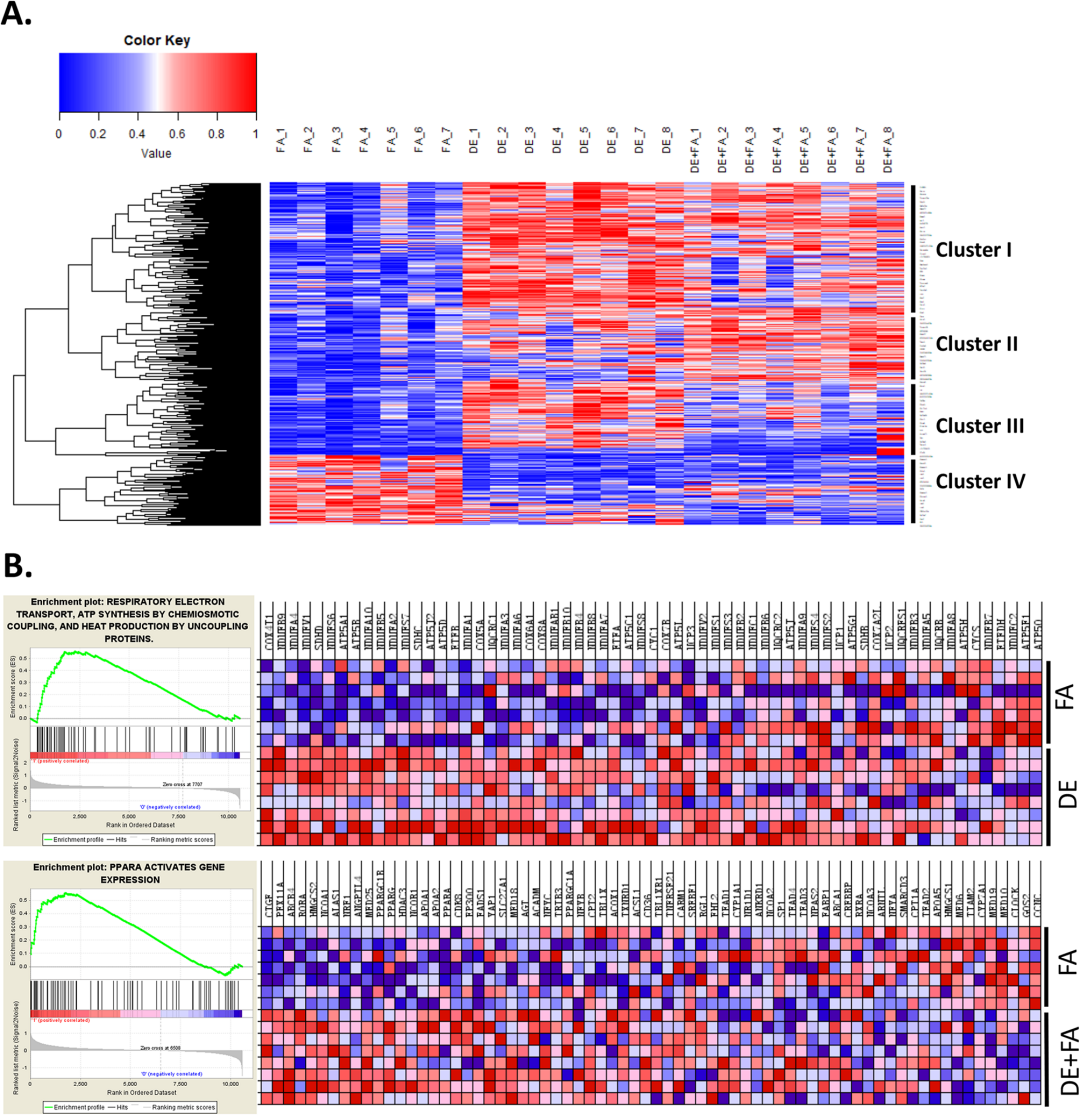
DE induced extensive gene dysregulation. (**A**) Heatmap of all differentially expressed genes (DEGs) using FA as reference group. (**B**) Gene set enrichment analysis (GSEA) of DE and DE + FA shows dissimilar patterns of gene expression. Top panel shows the gene expression pattern and heatmap for genes involved in respiratory electron transport, and ATP synthesis in the DE group. Bottom panel shows the gene expression pattern and heatmap for genes involved in PPARα activates gene expression gene set in the DE + FA group

We have previously shown that DE exposures significantly upregulate the expression of several antioxidant genes and activate the 5-lipoxygenase pathway in the liver [7]. Here, global gene expression profiling of livers from mice exposed to either DE, FA or DE followed by FA in Protocol 1 (*n* = 7–8/group), enabled us to determine the large extent of DE-induced transcriptomic changes using Illumina microarray technology (Fig. 1A). Altogether, 658 DEGs showed significant changes in expression level (FDR < 0.05) by the DE and DE + FA exposures (Fig. 1B), with more upregulated than downregulated genes for both exposure conditions when compared to FA controls, as shown in the Venn diagrams (Fig. 1B) and heatmaps (Fig. 2A). The heatmap indicates markedly different patterns of expression, which for simplicity, could be allocated to 4 clusters. Thus, a large number of genes was upregulated by DE (clusters I to III) while a smaller number of genes was significantly downregulated instead (cluster IV). While some of the upregulated genes by DE exposure returned to levels similar to FA by the additional week of FA exposure (Cluster III), other genes displayed further upregulation in the DE + FA mice instead (Cluster II).

To identify key pathways altered by DE exposures, we performed gene set enrichment analysis (GSEA) on the DEG sets for DE and DE + FA conditions. GSEA revealed significant perturbation of pathways involving whole cell metabolism (lipid, carbohydrate, amino acid and bile acids), respiratory electron transport, circadian clock and activation of genes by ATF4, among others (Supplementary Fig. 1 and Supplementary Table 5). Differentially expressed genes that were involved in lipid metabolism and transport included 1-acylglycerol-3-phosphate O-acyltransferase 3 (Agpat3), 3-hydroxy-3-methylglutaryl-Coenzyme A synthase 2 (Hmgcs2), ATP binding cassette subfamily G members (Abcg5 and Abcg8), ATP-binding cassette, sub-family B member 4 (Abcb4), acetyl-Coenzyme A acetyltransferase 1 (Acat1), acyl-Coenzyme A dehydrogenase, short chain (Acads) and adiponectin receptor 1 (Adipor1). These genes were upregulated in livers from DE exposed mice compared to FA controls (Fig. 2A). The concomitant increase in Abcg5 and Abcg8 indicate increased transport of cholesterol out of hepatocytes, possibly as a protective response to prevent net accumulation of sterols [30].

Significantly, DE-altered genes also included mitochondrial membrane transporters or the solute carrier family 25 such as Slc25a12, Slc25a10, Slc25a25, Slc25a28 and Slc25a39. Notably, genes involved in the respiratory electron transport pathways such as NADH ubiquinone oxidoreductase complex subunits (NDUFA) and ATPase H + transporting subunits (ATP1a1), showed significant upregulation and enrichment in the DE group compared to the FA controls (Fig. 2B, upper panel). Livers from the DE + FA exposed mice also showed significant perturbation of pathways involving similar processes and interestingly, nuclear receptors transcription pathways were among the top dysregulated pathways (Supplementary Table 5). Indeed, the PPARα activated gene expression pathway was significantly enriched in the DE + FA group (Fig. 2B, lower panel). We also performed pathway analysis using the Molecular Signature Database (MSigDB) which is one of the largest repositories of curated and annotated genes sets. As noted in Table 2, key metabolic processes involving lipids and the respiratory chain were significantly enriched.

**Table 2:** DE exposure alters multiple pathways in the liver

Gene Set Name	*p*-value	FDR q-value	Enrichment
Metabolism of lipids and lipoproteins	8.47E-14	1.30E-10	6.5
Lysosome	3.67E-11	8.72E-09	12.8
Transcription	8.02E-11	1.69E-08	4.4
Fatty acid triacylglycerol and ketone body metabolism	2E-10	3.81E-08	9.9
Response to stress	5.51E-10	8.73E-08	5.2
Negative regulation of programmed cell death	5.97E-10	8.73E-08	10.3
RNA metabolic process	9.56E-10	1.30E-07	4.0
Oxidative phosphorylation	1.9E-09	2.26E-07	10.6
Metabolism of amino acids and derivatives	1.82E-08	1.38E-06	7.7
TCA cycle and respiratory electron transport	3.62E-07	2.09E-05	8.4
Cellular carbohydrate metabolic process	1.32E-06	6.25E-05	8.5
Valine, leucine and isoleucine degradation	1.82E-06	7.87E-05	16.2
PPARA activates gene expression	2.94E-06	1.19E-04	9.2
Caraboxylic acid metabolic process	3.03E-06	1.2E-04	6.7

The table shows the 14 top gene pathways significantly affected by DE exposures using the GSEA/MSigDB, *p* < 0.05, FDR < 0.05

### DE exposure induces widespread changes in liver metabolites

**Fig. 3:**
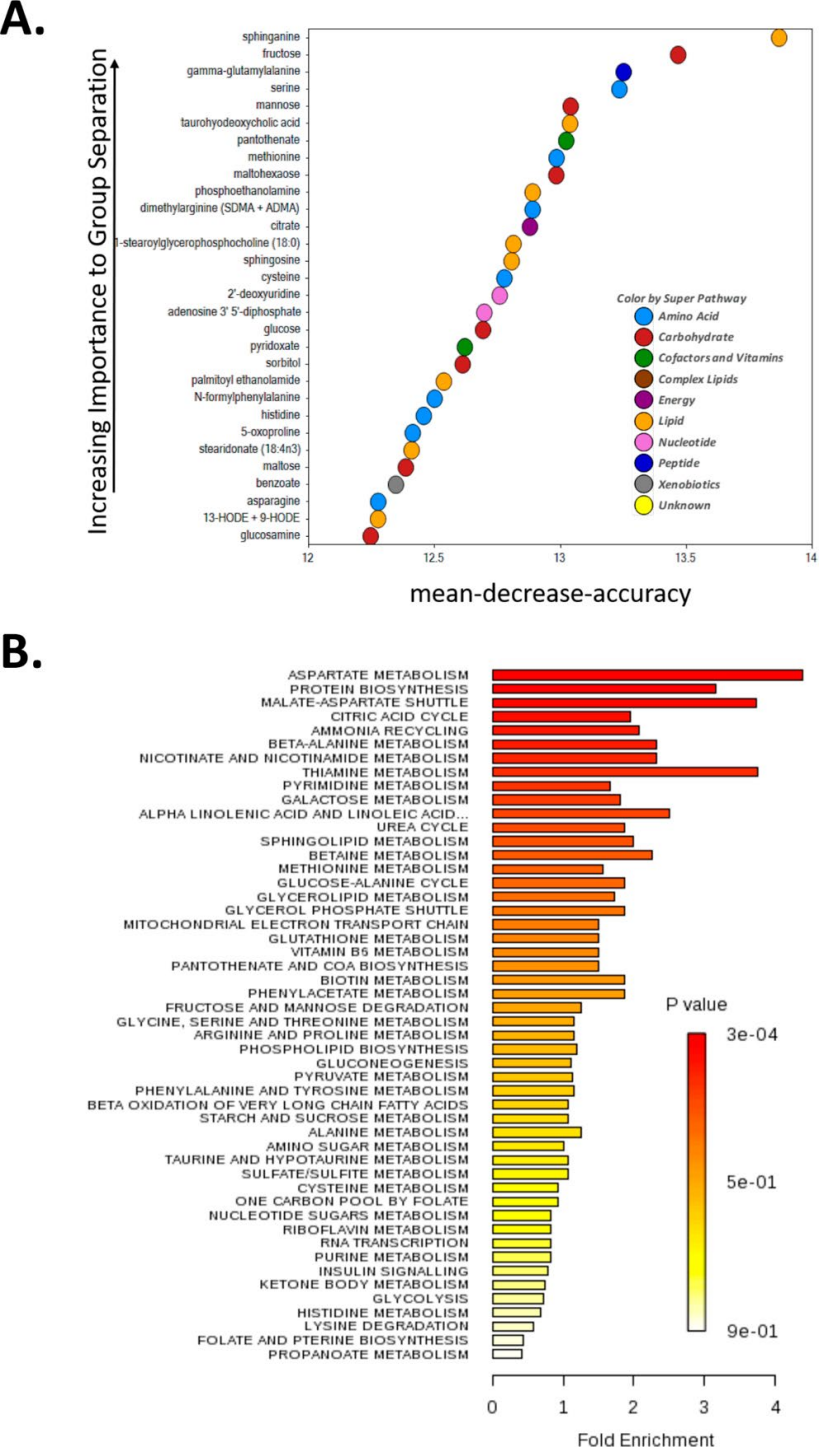
Metabolomic Analyses. (**A**) Random Forest Biochemical Importance Plot from liver. (**B**) Enrichment of several pathways as determined from metabolomics using KEGG pathway analyses

We conducted metabolomic analysis of liver samples from FA or DE-exposed mice for 2 weeks in Protocol 2, to gain an understanding of the nature of metabolic changes taking place in the liver and its involvement in inducing systemic metabolic effects. Comparison of global biochemical hepatic profiles revealed several key metabolic differences between DE and FA groups. The metabolomic analysis identified 70 metabolites that were significantly upregulated in the DE group while 48 metabolites were downregulated (Table 3) at FDR < 0.1. Random Forest was able to classify between the FA and DE exposure groups with ~ 90% accuracy based on metabolomic profiles. The metabolites key to classifying the two exposure groups included compounds associated with glucose and glycogen metabolism as well as markers of lipid peroxidation – 13-HODE and 9-HODE (Fig. 3A). Other oxidative stress responses included upregulation of genes and metabolites involved in glutathione metabolism (Supplementary Fig. 2).

**Table 3:** Summary of metabolomics analysis of FA and DE exposed livers

Two-Sample t-Test	Diesel Exhaust Filtered Air
	Metabolome
Total biochemical, FDR adjusted *p* ≤ 0.05	118
Increased biochemicals	70
Decreased biochemicals	48

We performed pathway analysis of the significantly altered metabolites and revealed significant enrichment in whole cell metabolic pathways involving lipid, carbohydrate, protein, and vitamin metabolism (Fig. 3B). Processes occurring in or involving mitochondria were particularly affected, such as the citric acid cycle, the malate-aspartate and glycerol phosphate shuttles, the mitochondrial electron transport and beta oxidation of very long chain fatty acids (Fig. 3B). These results are consistent with those observed in the transcriptome-based analysis in Protocol 1, and with the changes we previously observed in the mitochondrial function of HepG2 cells treated with DEP [12].

**Fig. 4:**
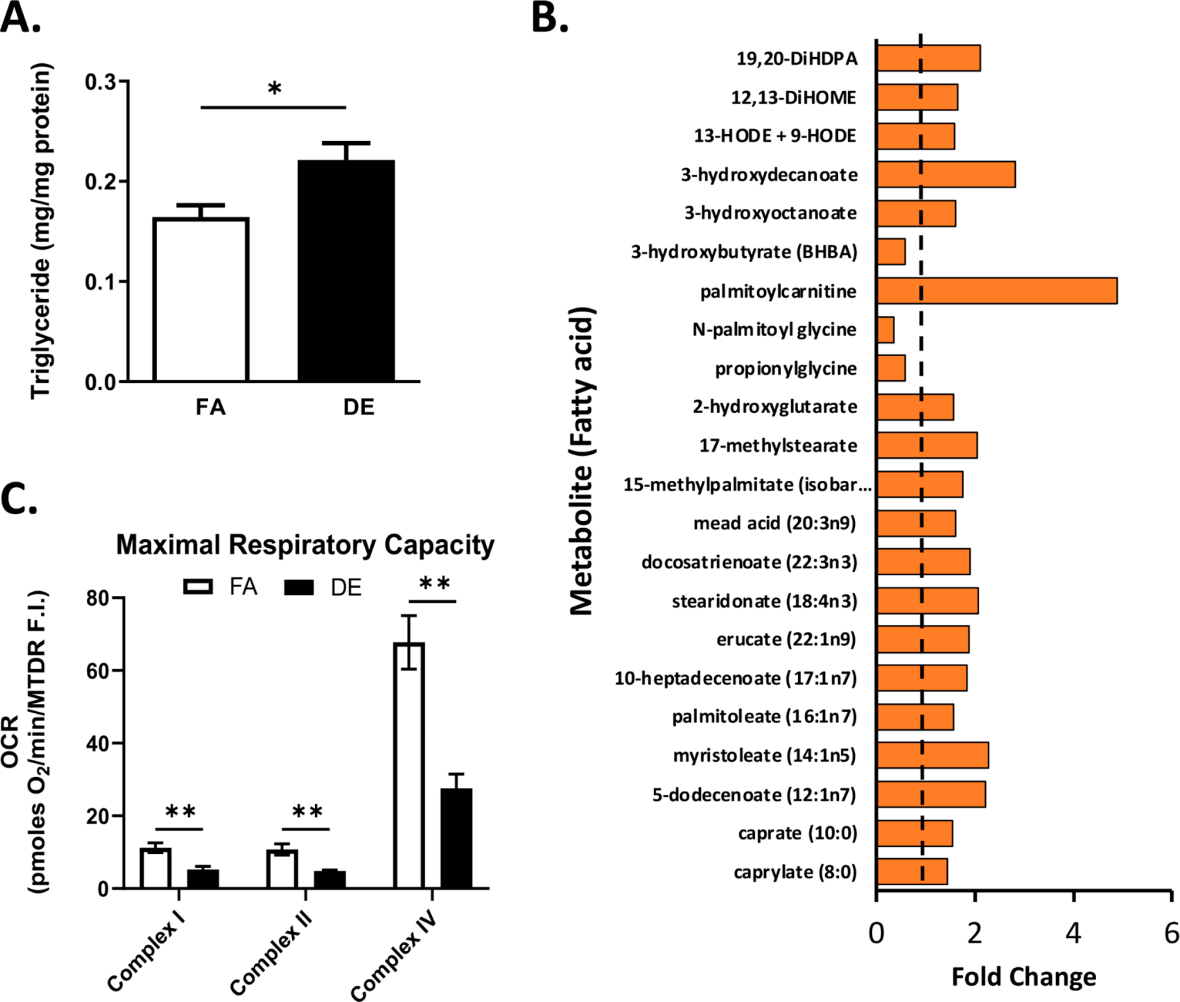
Hepatic Triglycerides, Fatty Acids and Mitochondrial Respiration. (**A**) The level of hepatic triglycerides (TG) was increased in the livers of 2-week DE-exposed ApoE^−/−^ mice compared to the FA group (Protocol 1). (**B**) Increased levels of fatty acids in the liver of DE exposed mice (Protocol 2). (**C**) Maximal respiratory capacity of mitochondrial complexes I, II and IV as determined by the oxygen consumption rate (OCR) in frozen livers of mice exposed to FA or DE for 2 weeks (Protocol 2). Statistical analysis was performed using Student t-test (**A**) or Mann-Whitney U test (**C**), *p < 0.05, **p < 0.01, DE vs. FA

We also observed here that only 2-week exposure to DE led to triglyceride accumulation (Fig. 4A) as well as increased levels of a large number of fatty acids in the liver (Fig. 4B). Importantly, DE exposure induced mitochondrial dysfunction in these mouse livers as determined by decreased oxygen consumption rate (OCR) in frozen liver samples from DE exposed mice as compared with FA controls (Fig. 4C). As previously reported, maximal respiratory capacity measured in frozen tissue is comparable to that in fresh tissue [31, 32]. Although freeze-thawing impairs mitochondrial respirations by breaking down the mitochondrial inner membrane, the interaction of electron transport complexes remains intact even with freeze-thaw cycles [31], enabling the assessment of electron transport chain function in vivo by determining it in frozen tissue. DE exposure was found to inhibit complex I function as measured by NADH substrate, complex II as measured by succinate and rotenone and complex IV activity as measured by TMPD + ascorbate, with the Seahorse assessment of OCR rates normalized by mitochondrial content measured via MTDR fluorescence (Fig. 4C). Interestingly, livers from DE-exposed mice exhibited increased MTDR fluorescence intensity (Supplementary Fig. 3) suggesting the possibility of increased mitochondrial content which together with decreased respiration could be due to accumulation of dysfunctional mitochondria. Our data is consistent with the increased triglyceride content noted in ApoE KO mice after 16-week exposure to DE, and mitochondrial dysfunction induced by a methanol extract of DEP on HepG2 cells and isolated mitochondria [12].

### Coordinated transcriptomic and metabolomics alterations in metabolic pathways

**Fig. 5:**
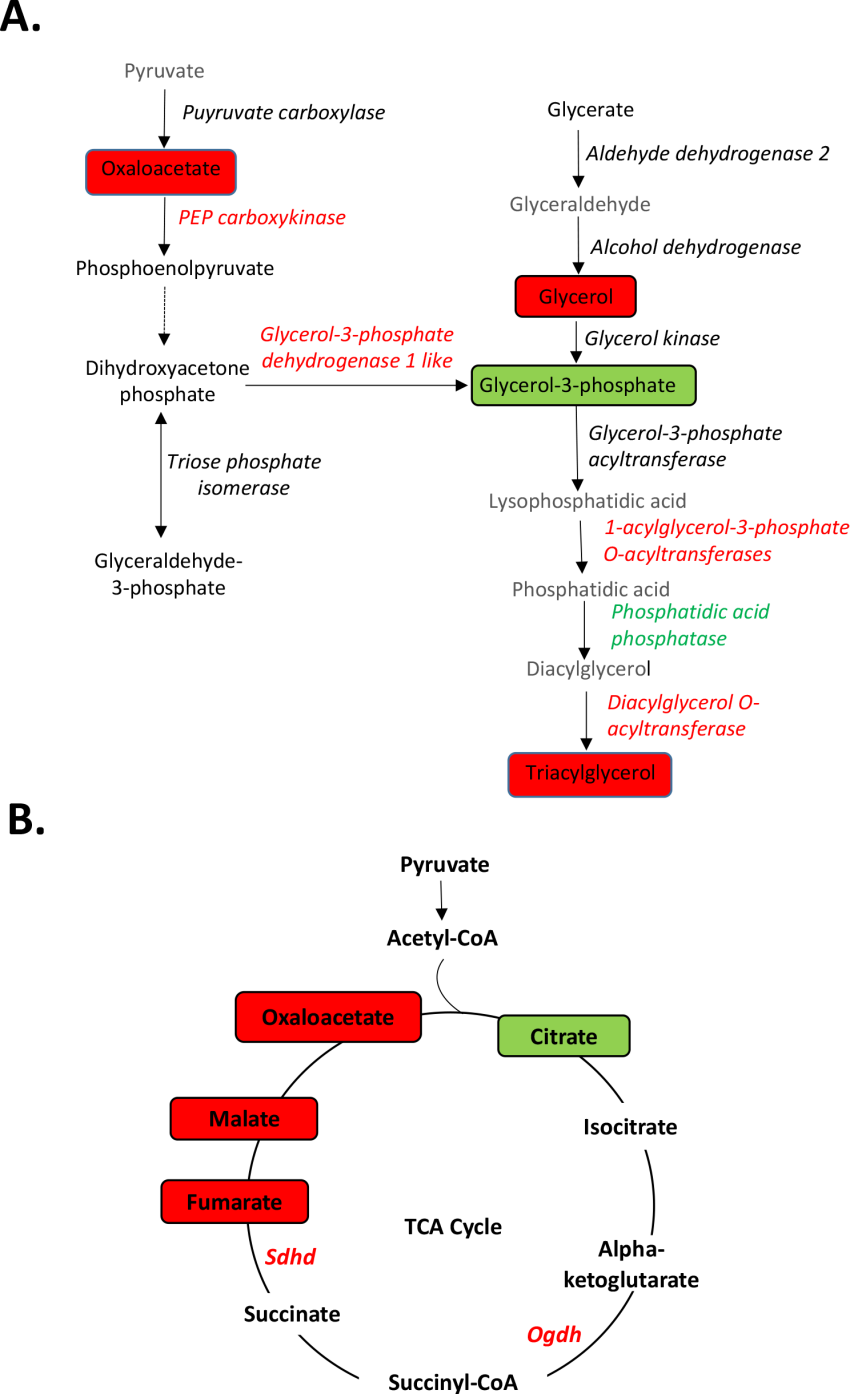
Integrated transcriptomic and metabolomic data. (**A**) Triacylglycerol synthesis and (**B**) TCA cycle. Pathways show the upregulated (red) or downregulated (green) metabolites (rectangles) and genes (italicized)

The omics data allowed us to gain an understanding on the early molecular changes occurring after only 2 weeks of DE exposure that could be responsible for promoting further long-term metabolic dysfunctions. We sought a composite view of the transcriptomic and metabolomic data by constructing pathways with converging signals from both approaches. As shown in Fig. 5A, the glycerolipid synthesis pathway showed changes in both metabolites and genes encoding enzymes that carry out the reactions. Specifically, the levels of glycerol and triacyglycerol were significantly increased in the livers of DE exposed mice while the intermediate glycerol-3-phosphate was decreased, likely due to increased usage. There was increased expression of genes encoding several enzymes including glycerol-3-phosphate dehydrogenase 1 like, 1-acylglycerol-3-phosphate O-acyltransferases and diacylglycerol O-acyltransferase (Fig. 5A), which could explain the triglyceride accumulation, especially in the context of increased levels of several fatty acids (Fig. 4B).

Our integrated analyses also showed a major perturbation in the citric acid cycle (Fig. 5B) which could mediate fatty acid accumulation likely due to decreased fatty acid beta-oxidation. This was reflected by a marked decrease (~ 3 fold) in the levels of citrate, significant increases in oxaloacetate (~ 2.5 fold), malate (~ 1.6 fold) and fumarate (~ 1.6 fold) levels (Table 4; Fig. 5B), and a significant, ~ 4-fold increase in reduced NAD (NADH) levels in DE exposed mice compared to controls (Table 4).

**Table 4:** Changes in levels of key metabolites involved in the tricarboxylic acid (TCA) cycle

Metabolite	Fold change	*p*-value	q-value
Citrate	0.35	2.0E-04*	8.5E-03
Alpha-ketoglutarate	1.77	1.68E-01	2.12E-01
Succinate	0.99	9.42E-01	5.26E-01
Fumarate	1.65	2.3E-02*	8.97E-02
Malate	1.66	1.42E-02*	7.38E-02
Oxaloacetate	2.33	3.98E-02*	1.12E-01
Nicotinamide adenine dinucleotide reduced (NADH)	4.18	2.24E-02*	8.96E-02

*FDR adjusted p-value < 0.05. TCA, tricarboxylic acid

### Gene network analysis identifies Pck1 as a key regulator for DE-induced changes in carbohydrate metabolism

**Fig. 6:**
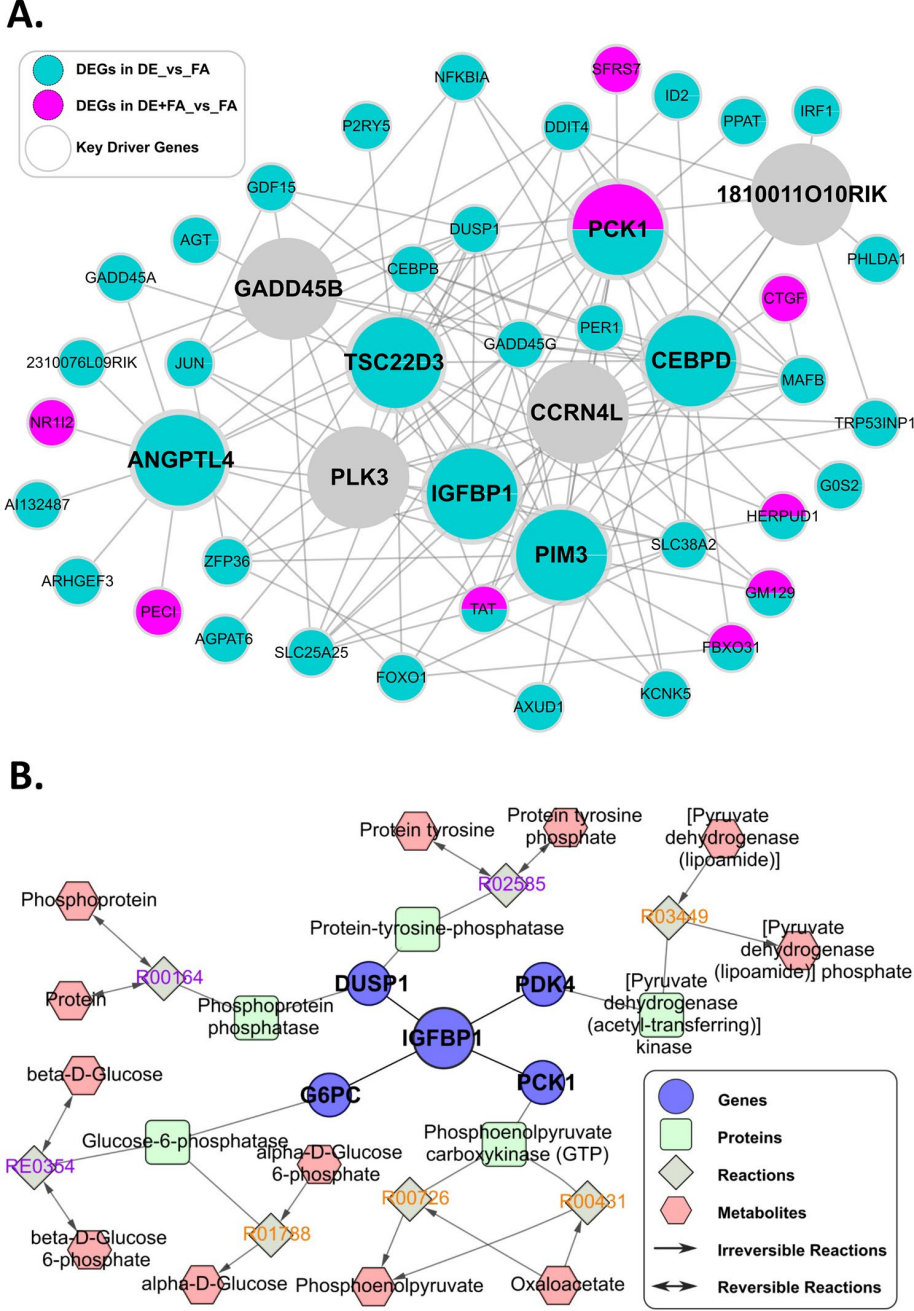
Gene Signatures. (**A**) Key driver analysis shows key driver genes (large circles) and member genes (small circles) of the DE (turquoise) and DE + FA (pink) gene signatures. (**B**) Metabolic reactions related to selected key driver genes and metabolites

To identify central regulators for DE effects and gene–gene interactions within the DE gene signature, we employed data-driven Bayesian networks (BNs) that elucidate gene–gene regulatory relationships (detailed in Materials and Methods). Using these networks and a network topology-based KDA, we identified multiple candidate key driver (KD) genes whose network neighboring genes highly overlapped with the DE signature genes. Some of the key driver genes identified included *Pck1*, *Pim3*, *Igfbp1*, *Tsc22d3*, *Angptl4* and *Ccrn4l*. Pim3 is a tyrosine kinase and is involved in tumorigenesis, but there is also evidence that is contributes to adipogenesis and glucose homeostasis [33]. *Igfbp1* is also a known regulator of glucose metabolism [34]. *Tsc22d3* encodes the protein glucocorticoid-induced leucine zipper which has anti-inflammatory and immunosuppressive actions [35]. Interestingly, *Angptl4* is involved in triglyceride metabolism by inhibiting lipoprotein lipase and increasing circulating triglyceride levels [36]. *Crn4l* or nocturnin (*Noc*) is a key circadian clock gene that is implicated in lipid metabolism, adipogenesis and glucose homeostasis [37]. As shown in Fig. 6A, *Pck1*, a major regulator of gluconeogenesis, was the only key driver gene for both DE and DE + FA groups, surrounded by many gene signatures. The identification of *Pck1* as one of the KDs was consistent with “cellular carbohydrate metabolic process” (Table 2) as significantly dysregulated and enriched pathway in the transcriptomic analyses. In addition, our metabolomics data revealed a large increase in the breakdown products of glycogen namely maltose and maltohexaose suggestive of increased glycogenolysis (Table 5).

**Table 5:** Levels of glycogen breakdown products in DE group compared to FA controls

Metabolite	Fold change	*p*-value	q-value
Maltohexaose	104.13	5.0E-04*	1.41E-02
Maltopentaose	1.72	1.07E-01	1.76E-01
Maltotetraose	1.41	8.98E-02	1.65E-01
Maltotriose	1.13	1.22E-01	1.89E-01
Maltose	18.13	1.4E-03*	2.55E-02
Fructose	9.6	3.05E-07*	9.16E-05
Sorbitol	3.6	2.27E-02*	8.96E-02
Mannose	4.52	1.07E-05*	1.2E-03
Mannose-6-phosphate	4.35	2.68E-01	2.87E-01
Tagatose	2.46	3.44E-02*	1.10E-01
Galactose 1-phosphate	4.67	5.9E-03*	5.04E-02
Galactonate	1.49	5.65E-01	4.08E-01

*p-value < 0.05

Since liver glucose was also significantly increased upon DE exposure, 3.26-fold, we investigated gluconeogenesis as a possible cellular carbohydrate metabolic process that contributes to this increase. Phosphoenolpyruvate carboxykinase (Pck1) was found to be a significantly upregulated gene in both the DE and DE + FA groups. Additional carbohydrate pathways such as glycolysis, gluconeogenesis, galactose metabolism, mannose metabolism, polyol pathway and glucosamine pathway also showed coordinated alterations in transcriptomic and metabolic signals (Supplementary Figs. 1, 2 and 4). Based on the KDA results and metabolomics data, we curated an integrative map of transcriptome and metabolome centered on the *Pck1* gene and glucose (Fig. 6B).

**Fig. 7:**
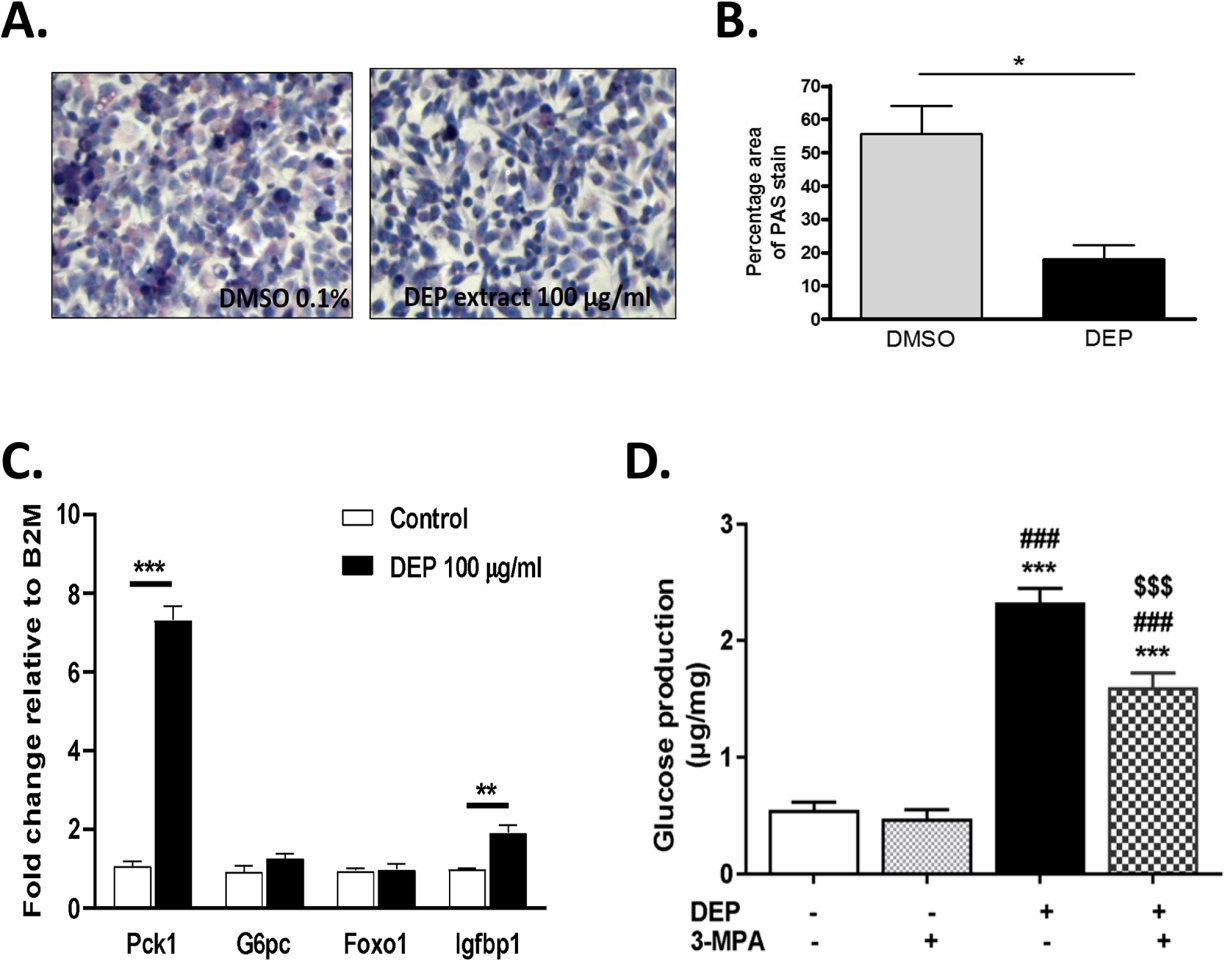
DEP stimulates glycogenolysis and gluconeogenesis. Diesel exhaust particulate (DEP) induces glycogenolysis in HepG2 cells as noted by (**A**) periodic acid Schiff (PAS) staining and (**B**) quantification of PAS stain using ImageJ software. (**C**) mRNA expression of PCK1 in HepG2 cells treated with DEP for 8 h. (**D**) Glucose production. Inhibition of PCK1 by 3-mercaptopicolinic acid (3-MPA) decreases DEP-induced glucose production. HepG2 cells were treated with DEP for 8 h in the presence or absence of 3-MPA. Data are shown as mean ± SEM, *p < 0.05, **p < 0.01 and ***p < 0.001, DEP vs. DMSO control group using student’s t-test. For panel (**D**), data was analyzed using one-way ANOVA followed by Tukey’s post hoc test, ***p<0.001, vs. DMSO control group; ^###^p<0.001, vs. 3-MPA treatment, ^$$$^p<0.001, vs DEP treament

To evaluate our network KD prediction and validate transcriptomic and metabolomics findings, we tested the effects of a methanol extract of DEP on HepG2 cells in vitro, without inducing any significant cytotoxicity in a concentration range of 10–100 µg/ml over 24 h of treatment as judged by the MTT assay (Supplementary Fig. 5). We observed a substantial decrease in glycogen content in cells treated with DEP extract compared to controls (Fig. 7A). Indeed, DEP-treated cells exhibited a significant decrease in the PAS-stained area, quantified with the use of the ImageJ software (Fig. 7B), indicative of increased glycogenolysis. DEP treatment also led to significant upregulation of *Pck1* and *Igfbp1* after 8 h by qPCR (Fig. 7C). To determine whether DEP-induced Pck1 led to increased gluconeogenesis, we first demonstrated significantly increased glucose production in DEP-treated HepG2 cells, compared to DMSO controls, at different times of treatment (8, 16 and 24 h) with DEP 100 µg/ml (Supplementary Fig. 6A) which was also replicated in primary hepatocytes treated with the DEP extract 25 µg/ml for 8 h (Supplementary Fig. 6B). We then demonstrated that DEP-induced glucose production was due to increased *Pck1* expression and functional activity as addition of Pck1 enzymatic inhibitor, 3-Mercaptopicolinic acid (3-MPA), significantly abrogated increased glucose production induced by DEP (Fig. 7D). These in-vitro data extend the findings from the in-vivo work, supporting the identification of Pck1 as an important key driver, regulating increased glucose production by activation of the gluconeogenesis pathway.

## Discussion

Our integrated transcriptomic and metabolomic study has identified multiple molecular pathways dysregulated by short-term exposures to diesel pollutants resulting in widespread alterations of cell metabolism in the liver. We have employed a strategy consisting of three sequential tiers of experimentation and/or analyses. In the first tier, pathway analyses were performed on liver transcriptomic and metabolomic data from mice belonging to the various exposure groups to identify molecular pathways dysregulated by DE. In the second tier, we performed a network analysis to unveil candidate key driver genes responsible for the dysregulated pathways, and in the third tier, testing of candidate genes and pathways was performed in HepG2 cells, with validation in primary mouse hepatocytes. This strategy yielded novel contributions to the existing literature on the effects induced by DE exposure, including: (i) induction of mitochondrial dysfunction in-vivo with perturbation of the TCA cycle and diverse metabolic pathways involving carbohydrates, lipids, and proteins. (ii) activation of glycogenolysis and gluconeogenesis with possibly a metabolic futile cycle, (iii) Network-based identification of *Pck1* as a key player in the DE-induced dysregulation of gluconeogenesis. The findings shown in this work depict early metabolic changes which could progress to subclinical or overt clinical metabolic derangements when exposures are continued for a longer time.

Cumulative epidemiological and experimental studies indicate that air pollutants such as particulate matter, ozone and diesel exhaust lead to systemic metabolic derangement affecting glucose, lipid and protein metabolism in humans and rodents [4, 5, 7, 38–40] but the molecular mechanisms of these effects are largely unknown. We have focused our attention on the liver since it is the central organ regulating lipid, carbohydrate and protein metabolism as well as detoxifying responses. Through targeted much narrower analyses, we have previously reported that air pollutants exposure increases lipid peroxidation products of long chain fatty acids in humans and mice [12, 13], together with increased mRNA expression of antioxidant genes in the liver [7, 12]. In addition, air pollution has been linked to non-alcoholic fatty liver disease as long-term exposure to concentrated ambient PM causes liver inflammation, fibrosis and activation of insulin signaling pathways [4, 5]. In the current study, we aimed to characterize the molecular responseof ApoE^−/−^ mice to DE exposure more comprehensively by using liver transcriptomic and metabolomics approaches to dissect mechanisms responsible for global metabolic alterations. Indeed, we found that DE induced marked and broad alterations of multiple pathways involving mitochondrial functions, fatty acids, triacylglycerol and cellular carbohydrate metabolic processes, among many others. Interestingly, expression of lipid transporters such as Abcg5, Abcg8 and Abcb4 were altered in DE-exposed livers, indicating DE-induced alterations in lipid and cholesterol metabolism. Mitochondrial specific genes belonging to the mitochondrial solute carrier family (Slc25), the NADH: ubiquinone oxidoreductase mitochondrial complex genes (Ndufs6, Ndufv1, Ndufa1, Ndufa2, Ndufa4) and complex II of the respiratory chain, succinate dehydrogenase (Sdh1), were also significantly upregulated in the DE-group. These findings are likely to be mediated by mitochondrial dysfunction through DE exposure that is reflected in the significant accumulation of the reduced coenzyme NAD (NADH).

DE-exposed mice showed significant increases in the levels of the TCA cycle intermediates, fumarate, malate and oxaloacetate as well as a marked decrease in the level of citrate, suggesting a slowdown of the TCA cycle in the synthesis of citrate from oxaloacetate and acetyl CoA (Fig. 5B). This could be due to a concomitant increase of the reduced coenzyme NAD (NADH), which has been reported to inhibit citrate synthase [8]. The accumulation of NADH could result from increased glycolysis and/or impaired utilization of NADH in the electron transport chain. In addition, the electron transport chain can also be impaired in the setting of mitochondrial dysfunction. Indeed, the function of electron transport chain complexes I, II and IV were disrupted by DE exposure (Fig. 4C). We have used HepG2 cells treated with a methanol extract of DEP to model in-vitro, effects that had been observed in-vivo, to further dissect gene pathways and mechanisms mediating cardiometabolic toxicity induced by DE. This approach has succeeded since HepG2 cells treated with DEP extract exhibited impaired mitochondrial respiration and defective fatty acid oxidation [12], which is now confirmed in livers from DE exposed mice in the current work. It is very likely then that mitochondrial dysfunction led to fatty acid accumulation and increased triglyceride content in liver (Fig. 4A&B) in both short-term (like in this study) as well as long-term exposure to DE [12]. In addition, data from the metabolome profiling showed significant increases in the lipid oxidation products, 9-HODE + 13-HODE after 2-week DE exposure, which are consistent with our previous reports where 16-week DE exposure also led to increased 9- and 13-HODE in the liver [12], underlining an important role for the induction of oxidative stress responses. Thus, we have shown that DE induced several antioxidant genes in the liver such as the catalytic subunit of glutamate cysteine ligase (Gcl-c); NAD(P)H-quinone oxidoreductase 1 (Nqo-1) and superoxide dismutase 1 (Sod1) [7],regulated by the transcription factor Nrf2, considered a master regulator of the antioxidant response, consistent with our recent report that macrophage responses from 24 different strains of mice to DEP extract are dominated by antioxidant genes, regulated by Nrf2, which led macrophages to polarize into a Mox transcriptomic phenotype [41]. Importantly, we have recently reported that subacute exposures to air pollutants such as Bisphenol A associate with an altered triglyceride profile consisting in increases in saturated triglycerides, in healthy young adults traveling from less polluted Los Angeles to more pollutated Beijing for 10 weeks [42].

We also observed that carbohydrate metabolism was markedly altered by DE exposures. Specifically, glycogenolysis was affected leading to a heightened accumulation of glycogen breakdown products likely due to a stress response following DE exposure. Livers from DE-exposed mice had a 100-fold increase of maltohexaose and significant elevations of glucose and maltose (Table 5). Malto-oligosaccharides are associated with glycogen degradation, and several of these intermediates such as maltopentaose, maltotetraose and maltotriose, were present only in the DE samples and not in the FA group. Our data is consistent with the study of Zheng et al., where C57BL/6 mice exhibited a reduction of liver glycogen after exposures to PM _2.5_ for 10 weeks [5]. Although increased glycogenolysis and liver content of glucose did not translate into increased circulating plasma levels of glucose, it did lead to increased hepatic levels of sorbitol, fructose and glucosamine via activation of the polyol and glucosamine pathways (Supplementary Fig. 4).

Our network analysis identified central players in the dysregulatory effects of DE on cell metabolism, especially phosphoenolpyruvate carboxykinase (PEPCK-C or Pck1) as a top key driver in both DE and DE + FA groups. The *Pck1* gene encodes the cytosolic isoform of phosphoenolpyruvate carboxykinase, which is well-known for its function in gluconeogenesis from phosphoenolpyruvate (PEP) in the liver [43]. Overexpression of *Pck1* in mice results in excessive hepatic glucose production [44] while liver-specific *Pck1* knockout mice exhibit an inability to remove oxaloacetate from the Krebs cycle that leads to fasting induced fatty liver [43]. Therefore, these data suggested that DE promoted both glycogenolysis and gluconeogenesis, and the intriguing possibility of a futile cycle. Thus, we analyzed the effects of treating HepG2 cells with a methanol extract of DEP, which markedly stimulated glycogen degradation (Fig. 7A&B). Likewise, DEP extract led to upregulation of *Pck1* and increased glucose production when both HepG2 cells (Fig. 7C) and mouse primary hepatocytes (Supplementary Fig. 6B) were glucose starved. We demonstrated that increased glucose production was due to increased gluconeogenesis since inhibition of PCK1 enzymatic activity led to abrogation of PM-induced glucose production (Fig. 7D). Consistent with our results, Liu et al. have reported increased expression of *Pck1* in livers of mice exposed to PM_2.5_ for 6 months, suggesting a potential mechanism for PM-induced whole body insulin resistance [45]. In addition, other environmental toxins have been shown to increase Pck1 activity in the liver such as benzene [46], styrene [47] and cigarette smoke [48]. Interestingly, while the in-vivo data highly suggested that DE induced a futile cycle, we could not probe this with the in-vitro work, however, since glycolysis and gluconeogenesis were tested at different times and metabolic conditions.

It is likely that DE-induction of *Pck1* expression was due to increased oxidative stress in the liver, evidenced by increased hepatic levels of MDA, 5-HETE, 9-HODE and 13-HODE, after 2 weeks or 16 weeks exposure to DE [7, 12], and glutathione metabolism. Indeed, H4IIE hepatoma cells treated with buthionine sulfoximine, an inhibitor of glutathione synthesis also exhibit increased expression of Pck1 [49]. In addition, treatment of human hepatocytes with zinc oxide (ZnO) nanoparticles impact gluconeogenesis due to increased intracellular ROS production as well [50]. Interestingly, ZnO treatment also stimulated glycogenolysis as observed in our study. Glycogen breakdown is regulated by a complex interplay of protein phosphorylation/dephosphorylation and a decrease in ATP results in the breakdown of glycogen and the increased production of glucose 6-phosphate, one of the intermediaries of the glycolysis/gluconeogenesis pathway [51]. In addition, the upregulation of the lysosomal enzyme, acid alpha-glucosidase (Gaa) in the DE group (data not shown) which normally breaks down glycogen into glucose suggests glycogenolysis following a stress response. Similarly, arsenic treatment for 6 weeks significantly increased liver *Pck1* mRNA expression and decreased liver glycogen content in mice [52]. It is possible that pollutant-mediated oxidative stress and/or mitochondrial dysfunction could result in an energy imbalance leading to disruptions in hepatic carbohydrate metabolism that over long term can contribute to insulin resistance. In spite of this, DE exposure did not affect plasma levels of glucose, insulin or HOMA-IR, neither after 2-week exposure in the current study (Supplementary Fig. 7) or after 16-week exposure [12] even though those alterations have ensued in C57BL/6 mice exposed to PM_2.5_ [4, 5]. One possibility is that DE induced homeostatic adaptation responses that inhibited the development of subclinical or clinical alterations.

Although we demonstrate significant hepatic transcriptomic and metabolomic changes, especially in association with the lipid and carbohydrate processing and mitochondrial respiration in DE-exposed mice, our study has some limitations. First, our study design only included hyperlipidemic ApoE KO mice fed a chow diet since we aimed to identify pathways with the potential to mediate pro-atherogenic effects, and it will be important to determine if the findings observed here reproduce in normolipidemic C57BL/6 mice and determine whether the ApoE null mutation could function as a sensitizing background. Importantly, however, findings from our studies with ApoE KO mice have translated into humans as healthy individuals exposed to high levels of ambient pollutants for 6 to 8 weeks exhibit similar elevations in blood oxidative biomarkers [13] as those exhibited by ApoE KO mice exposed to DE for 2 weeks [53], indicating that these mice serve as an informative model to identify and dissect health effects induced by DE and PM in general. Second, studies were only performed in male mice despite the known sex bias in the air pollution-induced biological responses. Thus, future studies should include C57BL/6 mice fed a normal chow and a high fat diet as well as females to determine the interactions between DE exposures, normo vs. hyperlipidemic backgrounds and assess for differences between sexes.

In summary, we show that 2-week exposure to inhaled DE induced marked hepatic metabolic changes resulting in increased glycogenolysis, gluconeogenesis and triglyceride formation, together with prominent changes in liver transcriptome expression involving a multitude of processes including TCA cycle and glutathione metabolism among many others. Notably, PM-induced Pck1 could be at the intersection of effects on both carbohydrate and lipid metabolism. Thus, Pck1 is not only a key regulator of gluconeogenesis, but it is also involved in TAG synthesis by converting oxaloacetate to phosphoenolpyruvate. These changes support the involvement of a stress response in DE-induced hepatic metabolic impairment. Further studies will be required to determine the likely long-term consequences of these metabolic alterations, and whether targeting of Pck1 could represent a feasible strategy to inhibit metabolic effects induced by vehicle pollutants in the liver.

## Electronic supplementary material

Below is the link to the electronic supplementary material.

Supplementary Material 1

## Data Availability

The dataset supporting the conclusions of this article is available in the Mendeley Data repository, [https://data.mendeley.com/datasets/6c9ctnn2dg/1].

## Abbreviations

FA: Filtered Air
DE: Diesel Exhaust
DEP: Diesel Exhaust Particles
PM: Particulate Matter
ApoE: Apolipoprotein E
KDA: Key Driver Analysis
Pck1: Phosphoenolpyruvate carboxykinase
TCA: Tricarboxylic Acid

## References

[1] Brook RD, Rajagopalan S, Pope CA 3rd, Brook JR, Bhatnagar A, Diez-Roux AV, et al. Particulate matter air pollution and cardiovascular disease: an update to the scientific statement from the American Heart Association. Circulation. 2010;121(21):2331–78. 10.1161/CIR.0b013e3181dbece1 20458016

[2] Turner MC, Krewski D, Pope CA 3rd, Chen Y, Gapstur SM, Thun MJ. Long-term ambient fine particulate matter air pollution and lung cancer in a large cohort of never-smokers. Am J Respir Crit Care Med. 2011;184(12):1374–81. 10.1164/rccm.201106-1011OC 21980033

[3] Hsieh S, Leaderer BP, Feldstein AE, Santoro N, McKay LA, Caprio S et al. Traffic-related air pollution associations with cytokeratin-18, a marker of hepatocellular apoptosis, in an overweight and obese paediatric population. Pediatr Obes. 2017;13(6):342–7. 10.1111/ijpo.12228 PMC5775922 28730729

[4] Sun Q, Yue P, Deiuliis JA, Lumeng CN, Kampfrath T, Mikolaj MB, et al. Ambient air pollution exaggerates adipose inflammation and insulin resistance in a mouse model of diet-induced obesity. Circulation. 2009;119(4):538–46. 19153269 10.1161/CIRCULATIONAHA.108.799015 PMC3845676

[5] Zheng Z, Xu X, Zhang X, Wang A, Zhang C, Huttemann M, et al. Exposure to ambient particulate matter induces a NASH-like phenotype and impairs hepatic glucose metabolism in an animal model. J Hepatol. 2013;58(1):148–54. 22902548 10.1016/j.jhep.2012.08.009 PMC3527686

[6] Rajagopalan S, Brook RD. Air pollution and type 2 diabetes: mechanistic insights. Diabetes. 2012;61(12):3037–45. 23172950 10.2337/db12-0190 PMC3501850

[7] Yin F, Lawal A, Ricks J, Fox JR, Larson T, Navab M, et al. Diesel exhaust induces systemic lipid peroxidation and development of dysfunctional pro-oxidant and pro-inflammatory high-density lipoprotein. Arterioscler Thromb Vasc Biol. 2013;33(6):1153–61. 23559632 10.1161/ATVBAHA.112.300552

[8] Laing S, Wang G, Briazova T, Zhang C, Wang A, Zheng Z, et al. Airborne particulate matter selectively activates endoplasmic reticulum stress response in the lung and liver tissues. Am J Physiol Cell Physiol. 2010;299(4):C736–49. 20554909 10.1152/ajpcell.00529.2009 PMC2957267

[9] Li N, Sioutas C, Cho A, Schmitz D, Misra C, Sempf J, et al. Ultrafine particulate pollutants induce oxidative stress and mitochondrial damage. Environ Health Perspect. 2003;111(4):455–60. 12676598 10.1289/ehp.6000 PMC1241427

[10] Upadhyay D, Panduri V, Ghio A, Kamp DW. Particulate matter induces alveolar epithelial cell DNA damage and apoptosis: role of free radicals and the mitochondria. Am J Respir Cell Mol Biol. 2003;29(2):180–7. 12600817 10.1165/rcmb.2002-0269OC

[11] Araujo JA, Barajas B, Kleinman M, Wang X, Bennett BJ, Gong KW, et al. Ambient particulate pollutants in the ultrafine range promote early atherosclerosis and systemic oxidative stress. Circul Res. 2008;102(5):589–96. 10.1161/CIRCRESAHA.107.164970 PMC3014059 18202315

[12] Yin F, Gupta R, Vergnes L, Driscoll WS, Ricks J, Ramanathan G et al. Diesel Exhaust Induces Mitochondrial Dysfunction, Hyperlipidemia, and Liver Steatosis. Arteriosclerosis, thrombosis, and vascular biology. 2019;39(9):1776-86. 10.1161/ATVBAHA.119.312736 PMC6703953 31340670

[13] Lin Y, Ramanathan G, Zhu Y, Yin F, Rea ND, Lu X, et al. Pro-oxidative and proinflammatory effects after traveling from Los Angeles to Beijing: a biomarker-based natural experiment. Circulation. 2019;140(24):1995–2004. 31744317 10.1161/CIRCULATIONAHA.119.042054 PMC13392974

[14] Li R, Navab M, Pakbin P, Ning Z, Navab K, Hough G, et al. Ambient ultrafine particles alter lipid metabolism and HDL anti-oxidant capacity in LDLR-null mice. J Lipid Res. 2013;54(6):1608–15. 23564731 10.1194/jlr.M035014 PMC3646462

[15] Mootha VK, Lindgren CM, Eriksson KF, Subramanian A, Sihag S, Lehar J, et al. PGC-1alpha-responsive genes involved in oxidative phosphorylation are coordinately downregulated in human diabetes. Nat Genet. 2003;34(3):267–73. 12808457 10.1038/ng1180

[16] Subramanian A, Tamayo P, Mootha VK, Mukherjee S, Ebert BL, Gillette MA, et al. Gene set enrichment analysis: a knowledge-based approach for interpreting genome-wide expression profiles. Proc Natl Acad Sci USA. 2005;102(43):15545–50. 16199517 10.1073/pnas.0506580102 PMC1239896

[17] Shu L, Zhao Y, Kurt Z, Byars SG, Tukiainen T, Kettunen J, et al. Mergeomics: multidimensional data integration to identify pathogenic perturbations to biological systems. BMC Genomics. 2016;17(1):874. 27814671 10.1186/s12864-016-3198-9 PMC5097440

[18] Yang X, Deignan JL, Qi H, Zhu J, Qian S, Zhong J, et al. Validation of candidate causal genes for obesity that affect shared metabolic pathways and networks. Nat Genet. 2009;41(4):415–23. 19270708 10.1038/ng.325 PMC2837947

[19] Smoot ME, Ono K, Ruscheinski J, Wang PL, Ideker T. Cytoscape 2.8: new features for data integration and network visualization. Bioinformatics. 2011;27(3):431–2. 21149340 10.1093/bioinformatics/btq675 PMC3031041

[20] Yang X, Peterson L, Thieringer R, Deignan JL, Wang X, Zhu J, et al. Identification and validation of genes affecting aortic lesions in mice. J Clin Investig. 2010;120(7):2414–22. 20577049 10.1172/JCI42742 PMC2898611

[21] Zhao Y, Chen J, Freudenberg JM, Meng Q, Rajpal DK, Yang X. Network-based identification and prioritization of key regulators of coronary artery disease loci. Arterioscler Thromb Vasc Biol. 2016;36(5):928–41. 26966275 10.1161/ATVBAHA.115.306725 PMC5576868

[22] Li N, Kim S, Wang M, Froines J, Sioutas C, Nel A. Use of a stratified oxidative stress model to study the biological effects of ambient concentrated and diesel exhaust particulate matter. Inhal Toxicol. 2002;14(5):459–86. 12028803 10.1080/089583701753678571

[23] Li N, Alam J, Venkatesan MI, Eiguren-Fernandez A, Schmitz D, Di Stefano E, et al. Nrf2 is a key transcription factor that regulates antioxidant defense in macrophages and epithelial cells: protecting against the proinflammatory and oxidizing effects of diesel exhaust chemicals. J Immunol. 2004;173(5):3467–81. 15322212 10.4049/jimmunol.173.5.3467

[24] Gong KW, Zhao W, Li N, Barajas B, Kleinman M, Sioutas C, et al. Air-pollutant chemicals and oxidized lipids exhibit genome-wide synergistic effects on endothelial cells. Genome Biol. 2007;8(7):R149. 17655762 10.1186/gb-2007-8-7-r149 PMC2323217

[25] Lawal A, Zhang M, Dittmar M, Lulla A, Araujo JA. Heme oxygenase-1 protects endothelial cells from the toxicity of air pollutant chemicals. Toxicol Appl Pharmcol. 2015;284(3):281–91. 10.1016/j.taap.2015.01.010 PMC4743257 25620054

[26] Phalen RF, Oldham MJ, Nel AE. Tracheobronchial particle dose considerations for in vitro toxicology studies. Toxicol Sci. 2006;92(1):126–32. 16597657 10.1093/toxsci/kfj182

[27] Nemmar A, Hoet PH, Vanquickenborne B, Dinsdale D, Thomeer M, Hoylaerts MF, et al. Passage of inhaled particles into the blood circulation in humans. Circulation. 2002;105(4):411–4. 11815420 10.1161/hc0402.104118

[28] Gao R, Ku T, Ji X, Zhang Y, Li G, Sang N. Abnormal energy metabolism and tau phosphorylation in the brains of middle-aged mice in response to atmospheric PM2.5 exposure. J Environ Sci. 2017;62:145–53. 10.1016/j.jes.2017.06.037 29289286

[29] Charni-Natan M, Goldstein I. Protocol for primary mouse hepatocyte isolation. STAR Protoc. 2020;1(2):100086. 33111119 10.1016/j.xpro.2020.100086 PMC7580103

[30] Patel SB, Graf GA, Temel RE. ABCG5 and ABCG8: more than a defense against xenosterols. J Lipid Res. 2018;59(7):1103–13. 29728459 10.1194/jlr.R084244 PMC6027916

[31] Acin-Perez R, Benador IY, Petcherski A, Veliova M, Benavides GA, Lagarrigue S, et al. A novel approach to measure mitochondrial respiration in frozen biological samples. EMBO J. 2020;39(13):e104073. 32432379 10.15252/embj.2019104073 PMC7327496

[32] Osto C, Benador IY, Ngo J, Liesa M, Stiles L, Acin-Perez R, et al. Measuring mitochondrial respiration in previously frozen Biological samples. Curr Protoc Cell Biol. 2020;89(1):e116. 33320426 10.1002/cpcb.116

[33] Hepler C, Shan B, Zhang Q, Henry GH, Shao M, Vishvanath L et al. Identification of functionally distinct fibro-inflammatory and adipogenic stromal subpopulations in visceral adipose tissue of adult mice. Elife. 2018;7:e39636. 10.7554/eLife.39636 PMC6167054 30265241

[34] Lewitt MS, Denyer GS, Cooney GJ, Baxter RC. Insulin-like growth factor-binding protein-1 modulates blood glucose levels. Endocrinology. 1991;129(4):2254–6. 1717244 10.1210/endo-129-4-2254

[35] Ronchetti S, Migliorati G, Delfino DV. Association of inflammatory mediators with pain perception. Biomed Pharmacother. 2017;96:1445–52. 29217162 10.1016/j.biopha.2017.12.001

[36] Yoshida K, Shimizugawa T, Ono M, Furukawa H. Angiopoietin-like protein 4 is a potent hyperlipidemia-inducing factor in mice and inhibitor of lipoprotein lipase. J Lipid Res. 2002;43(11):1770–2. 12401877 10.1194/jlr.c200010-jlr200

[37] Stubblefield JJ, Gao P, Kilaru G, Mukadam B, Terrien J, Green CB. Temporal control of metabolic amplitude by Nocturnin. Cell Rep. 2018;22(5):1225–35. 29386110 10.1016/j.celrep.2018.01.011 PMC5815321

[38] Eze IC, Hemkens LG, Bucher HC, Hoffmann B, Schindler C, Kunzli N, et al. Association between ambient air pollution and diabetes mellitus in Europe and North America: systematic review and meta-analysis. Environ Health Perspect. 2015;123(5):381–9. 25625876 10.1289/ehp.1307823 PMC4421762

[39] Li R, Navab K, Hough G, Daher N, Zhang M, Mittelstein D, et al. Effect of exposure to atmospheric ultrafine particles on production of free fatty acids and lipid metabolites in the mouse small intestine. Environ Health Perspect. 2015;123(1):34–41. 25170928 10.1289/ehp.1307036 PMC4286268

[40] Li W, Dorans KS, Wilker EH, Rice MB, Long MT, Schwartz J, et al. Residential proximity to major roadways, fine particulate matter, and hepatic steatosis: the Framingham Heart Study. Am J Epidemiol. 2017;186(7):857–65. 28605427 10.1093/aje/kwx127 PMC5860476

[41] Bhetraratana M, Orozco LD, Bennett BJ, Luna K, Yang X, Lusis AJ et al. Diesel exhaust particle extract elicits an oxPAPC-like transcriptomic profile in macrophages across multiple mouse strains. Environ Pollut. 2024;358:124415. 10.1016/j.envpol.2024.124415 PMC13456106 38908672

[42] Lu X, Lin Y, Qiu X, Liu J, Zhu T, Araujo JA, et al. Triglyceride profiles are associated with subacute exposure to bisphenol A in healthy young adults. Sci Total Environ. 2022;825:153991. 35192814 10.1016/j.scitotenv.2022.153991 PMC13470991

[43] She P, Shiota M, Shelton KD, Chalkley R, Postic C, Magnuson MA. Phosphoenolpyruvate carboxykinase is necessary for the integration of hepatic energy metabolism. Mol Cell Biol. 2000;20(17):6508–17. 10938127 10.1128/mcb.20.17.6508-6517.2000 PMC86125

[44] Sun Y, Liu S, Ferguson S, Wang L, Klepcyk P, Yun JS, et al. Phosphoenolpyruvate carboxykinase overexpression selectively attenuates insulin signaling and hepatic insulin sensitivity in transgenic mice. J Biol Chem. 2002;277(26):23301–7. 11964395 10.1074/jbc.M200964200

[45] Liu C, Xu X, Bai Y, Zhong J, Wang A, Sun L, et al. Particulate Air pollution mediated effects on insulin resistance in mice are independent of CCR2. Part Fibre Toxicol. 2017;14(1):6. 28253935 10.1186/s12989-017-0187-3 PMC5335830

[46] Bahadar H, Maqbool F, Mostafalou S, Baeeri M, Gholami M, Ghafour-Boroujerdi E, et al. The molecular mechanisms of liver and islets of Langerhans toxicity by benzene and its metabolite hydroquinone in vivo and in vitro. Toxicol Mech Methods. 2015;25(8):628–36. 26056850 10.3109/15376516.2015.1053650

[47] Niaz K, Mabqool F, Khan F, Ismail Hassan F, Baeeri M, Navaei-Nigjeh M, et al. Molecular mechanisms of action of styrene toxicity in blood plasma and liver. Environ Toxicol. 2017;32(10):2256–66. 28678435 10.1002/tox.22441

[48] Neal RE, Chen J, Webb C, Stocke K, Gambrell C, Greene RM, et al. Developmental cigarette smoke exposure II: hepatic proteome profiles in 6 month old adult offspring. Reprod Toxicol. 2016;65:414–24. 27319396 10.1016/j.reprotox.2016.06.009 PMC5096382

[49] Ito Y, Oumi S, Nagasawa T, Nishizawa N. Oxidative stress induces phosphoenolpyruvate carboxykinase expression in H4IIE cells. Biosci Biotechnol Biochem. 2006;70(9):2191–8. 16960379 10.1271/bbb.60135

[50] Filippi C, Pryde A, Cowan P, Lee T, Hayes P, Donaldson K, et al. Toxicology of ZnO and TiO2 nanoparticles on hepatocytes: impact on metabolism and bioenergetics. Nanotoxicology. 2015;9(1):126–34. 24708275 10.3109/17435390.2014.895437

[51] Petersen MC, Vatner DF, Shulman GI. Regulation of hepatic glucose metabolism in health and disease. Nat Reviews Endocrinol. 2017;13(10):572–87. 10.1038/nrendo.2017.80 PMC5777172 28731034

[52] Huang CF, Yang CY, Chan DC, Wang CC, Huang KH, Wu CC, et al. Arsenic exposure and glucose Intolerance/Insulin resistance in estrogen-deficient female mice. Environ Health Perspect. 2015;123(11):1138–44. 25859628 10.1289/ehp.1408663 PMC4629734

[53] Yin F, Ramanathan G, Zhang M, Araujo JA. Prooxidative effects of ambient pollutant chemicals are inhibited by HDL. J Biochem Mol Toxicol. 2013;27(2):172–83. 23420698 10.1002/jbt.21475

