# Supplementary material for: Integrated hepatic transcriptomics and metabolomics identify Pck1 as a key factor in the broad dysregulation induced by vehicle pollutants
Source: Part Fibre Toxicol. 2024 Dec 30;21:55. doi: 10.1186/s12989-024-00605-6 (PMC11684268; doi:10.1186/s12989-024-00605-6)
Supplement: Supplementary file 1 — Supplementary Material 1 [file 12989_2024_605_MOESM1_ESM.pdf]

Supplementary Figures and Tables

Integrated Hepatic Transcriptomics and Metabolomics Identify Pck1 as a Key Factor in the Broad Dysregulation Induced by Vehicle Pollutants

Gajalakshmi Ramanathan[1], Yuqi Zhao[2], Rajat Gupta[1,3], Siri Langmo[1,3,4], May Bhetraratana[1], Fen Yin[1], Will Driscoll[5,6], Jerry Ricks[5,6], Allen Louie[1,3], James A. Stewart[6], Timothy R. Gould[7], Timothy V. Larson[6,7], Joel D. Kaufman[6], Michael E. Rosenfeld[5,6], Xia Yang[2,3,8], Jesus A. Araujo[1,3,4,8]

Corresponding author: Jesus A. Araujo, MD, PhD. Division of Cardiology, Department of Medicine, David Geffen School of Medicine, University of California-Los Angeles, 10833 Le Conte Avenue, CHS 43-264, Los Angeles, CA 90095. P.O. Box 951679. Phone number (310) 825-3222, Fax number (310) 206-9133. E-mail address: JAraujo@mednet.ucla.edu

A.

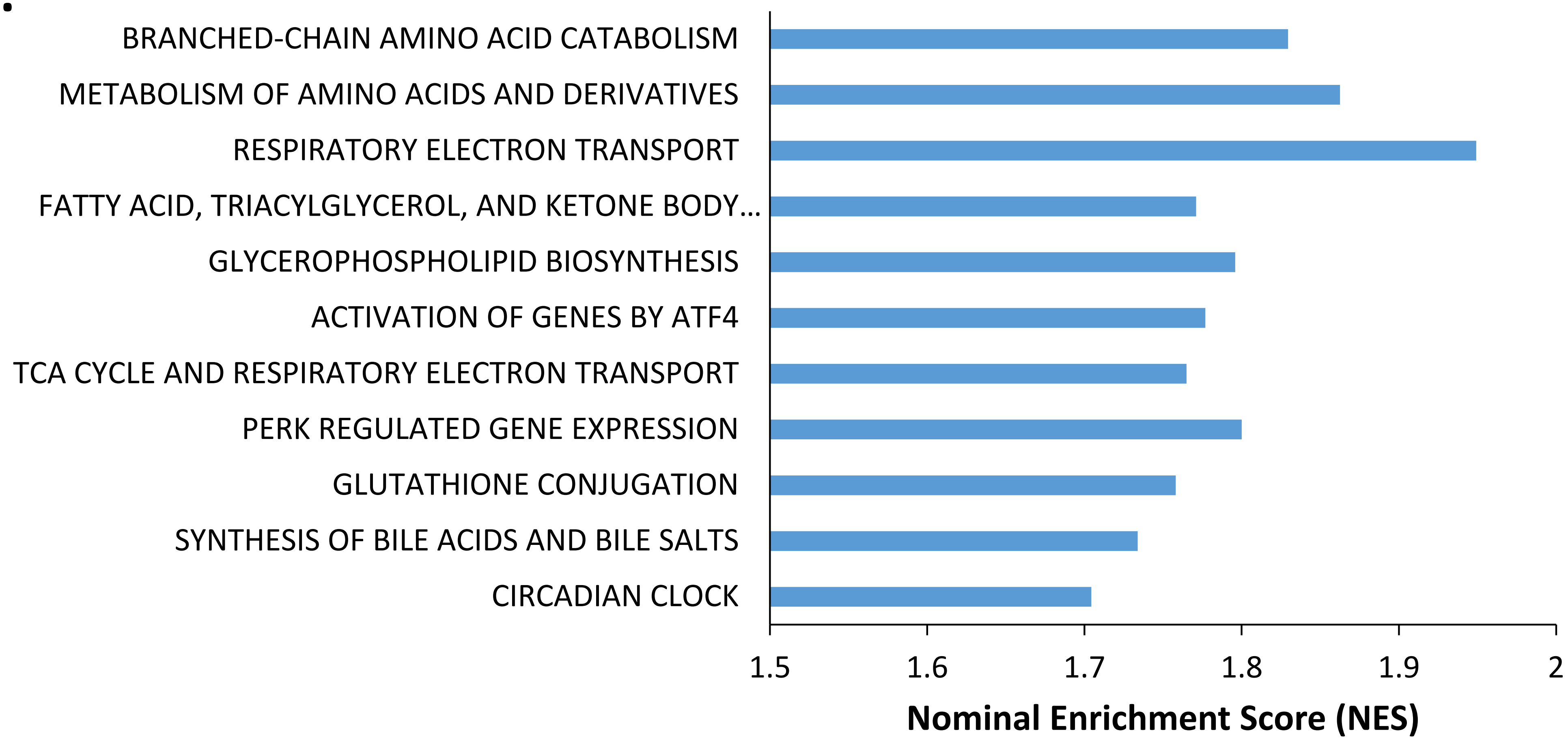

Supplementary Figure 1. Pathways regulated in the liver by DE exposure. Gene set enrichment analysis (GSEA) was performed on DEGs from **(A)** DE and **(B)** DE+FA groups. Nominal enrichment score (NES) for gene sets with p-value <0.05 and FDR<0.05 are shown.

B.

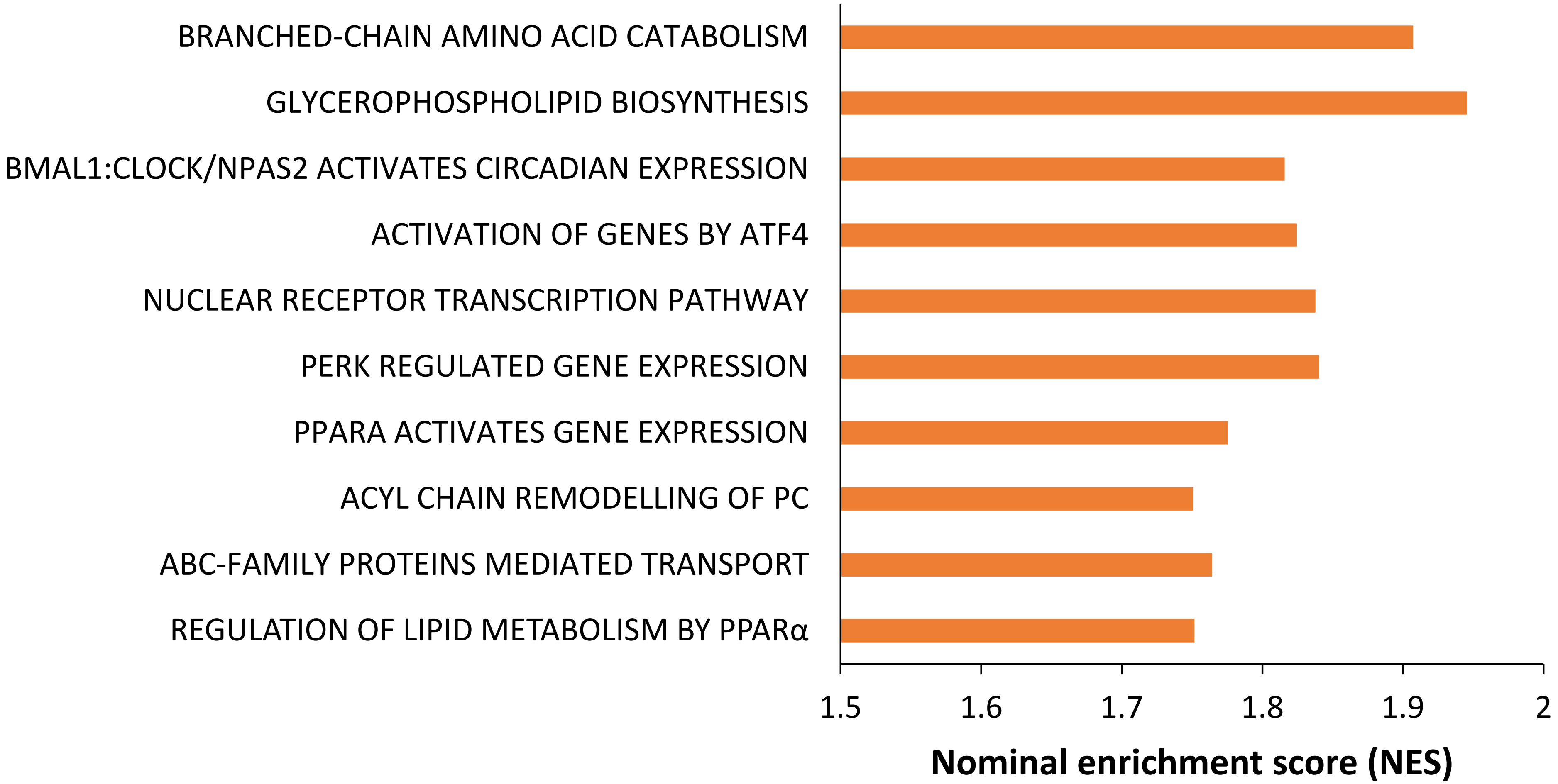

Supplementary Figure 1. Pathways regulated in the liver by DE exposure. Gene set enrichment analysis (GSEA) was performed on DEGs from **(A)** DE and **(B)** DE+FA groups. Nominal enrichment score (NES) for gene sets with p-value <0.05 and FDR<0.05 are shown.

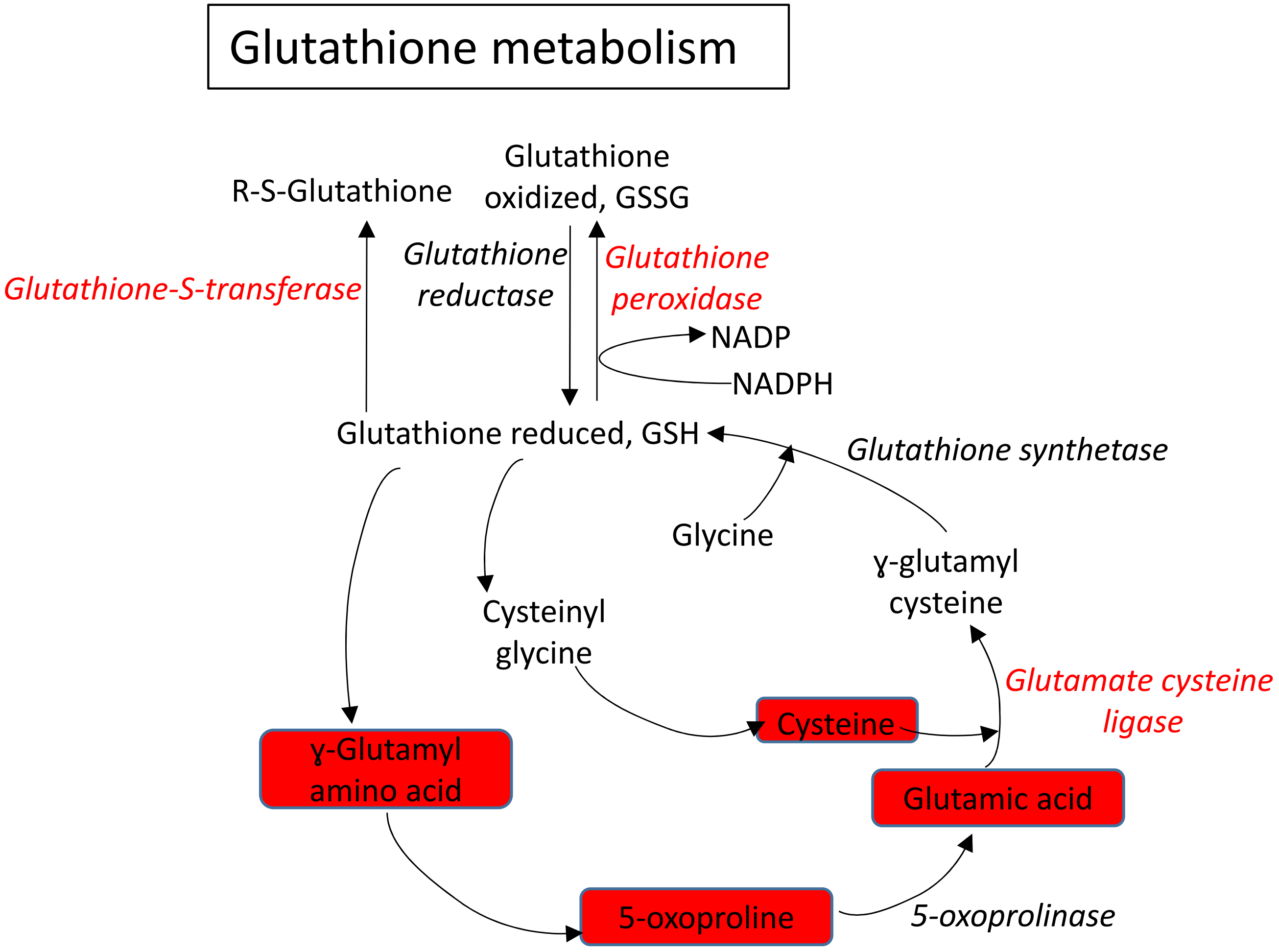

Supplementary Figure 2. Glutathione metabolism. Pathways show the upregulated (red) or downregulated (green) metabolites (rectangles) and genes (italicized).

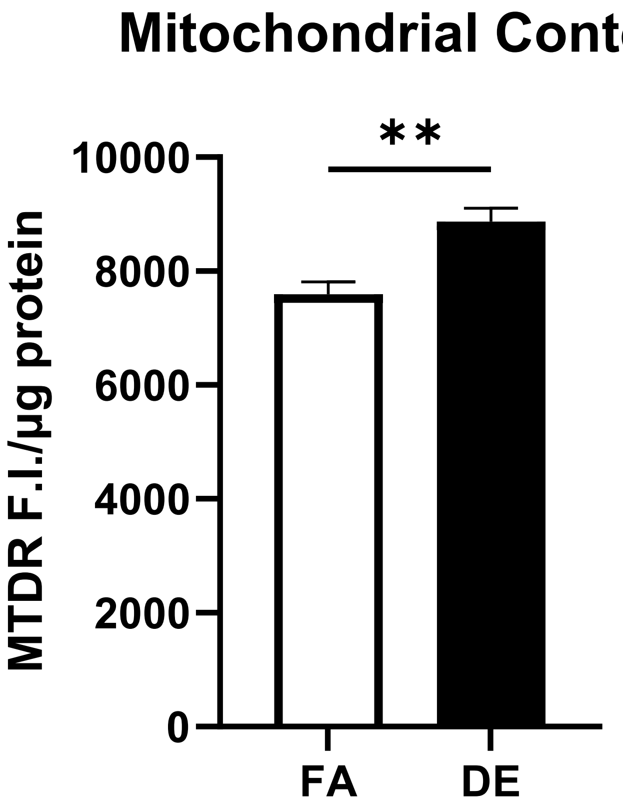

Supplementary Figure 3. Mitochondrial content was determined by MitoTracker Deep Red Fluorescence Intensity (MTDR F.I) in frozen liver tissues of mice exposed to DE (n=6) or FA (n=6) for 2 weeks. Values shown are mean ± SEM. Data was analyzed using unpaired student's t-test. **p<0.01, DE vs. FA control.

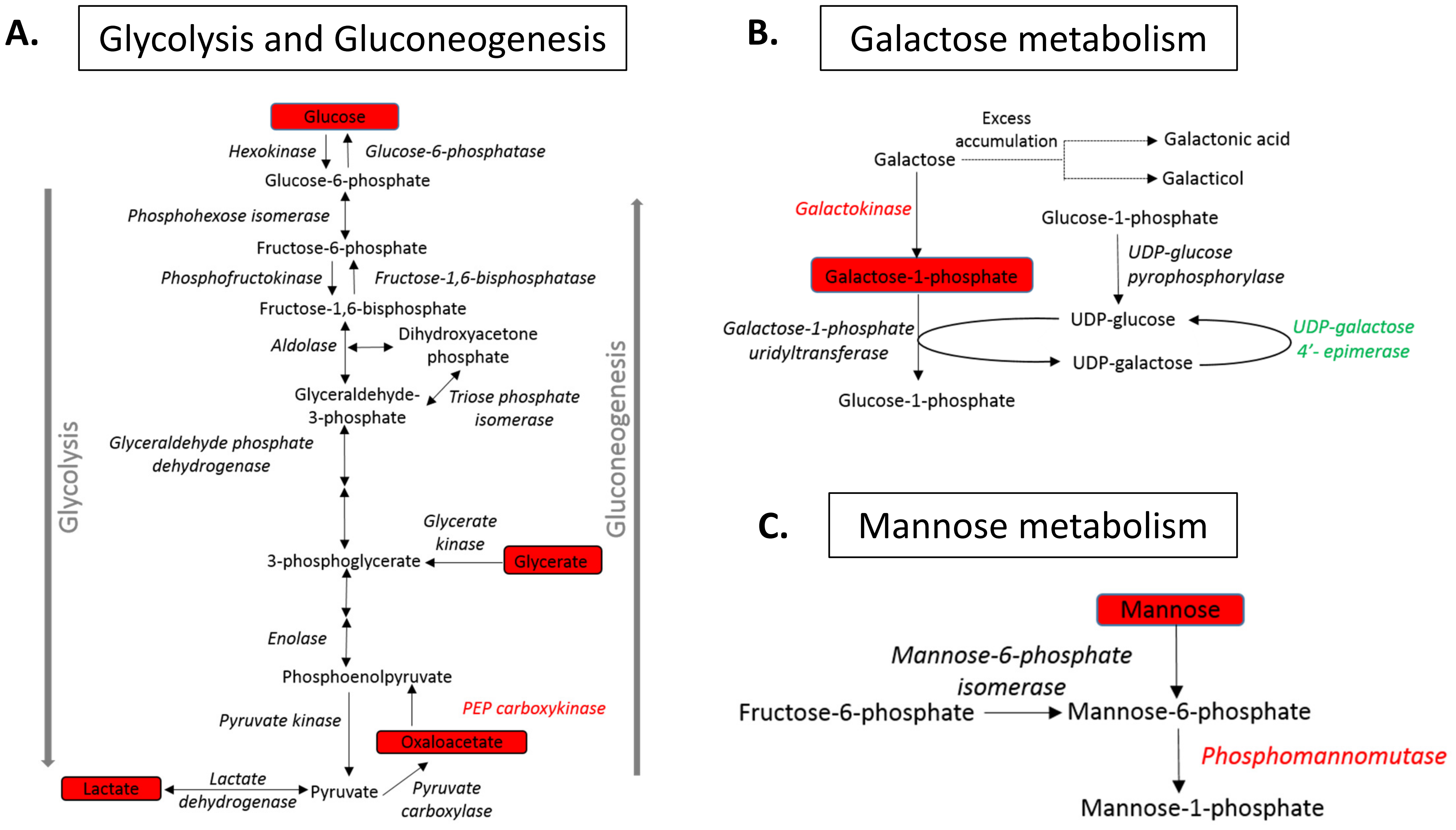

Supplementary Figure 4. (A) Glycolysis and gluconeogenesis, **(B)** galactose metabolism, **(C)** mannose metabolism, **(D)** polyol pathway and **(E)** glucosamine pathway. Pathways show the upregulated (red) or downregulated (green) metabolites (rectangles) and genes (italicized).

D. Polyol pathway

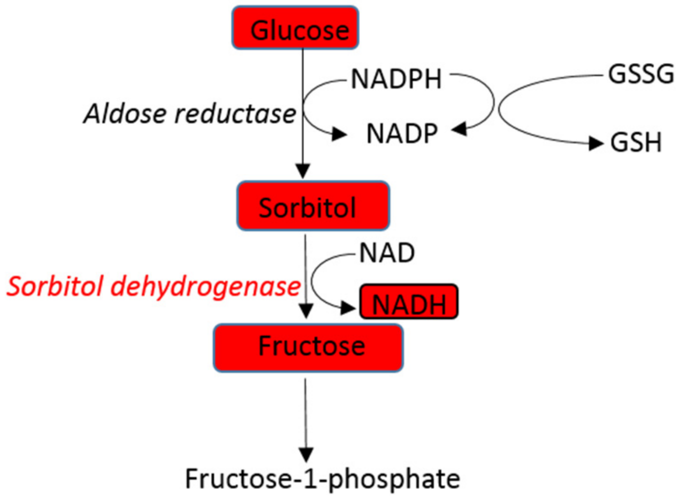

E. Glucosamine pathway

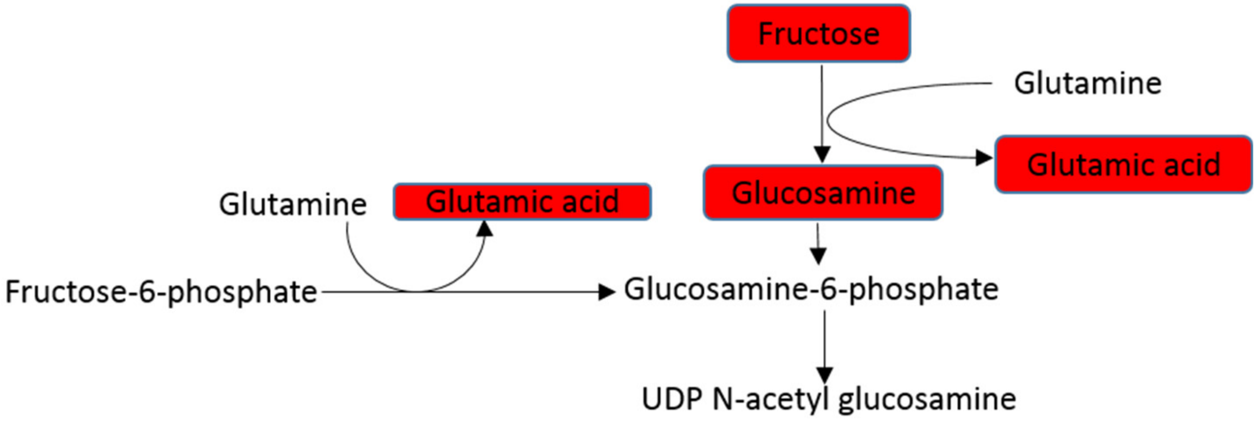

Supplementary Figure 4. (A) Glycolysis and gluconeogenesis, **(B)** galactose metabolism, **(C)** mannose metabolism, **(D)** polyol pathway and **(E)** glucosamine pathway. Pathways show the upregulated (red) or downregulated (green) metabolites (rectangles) and genes (italicized).

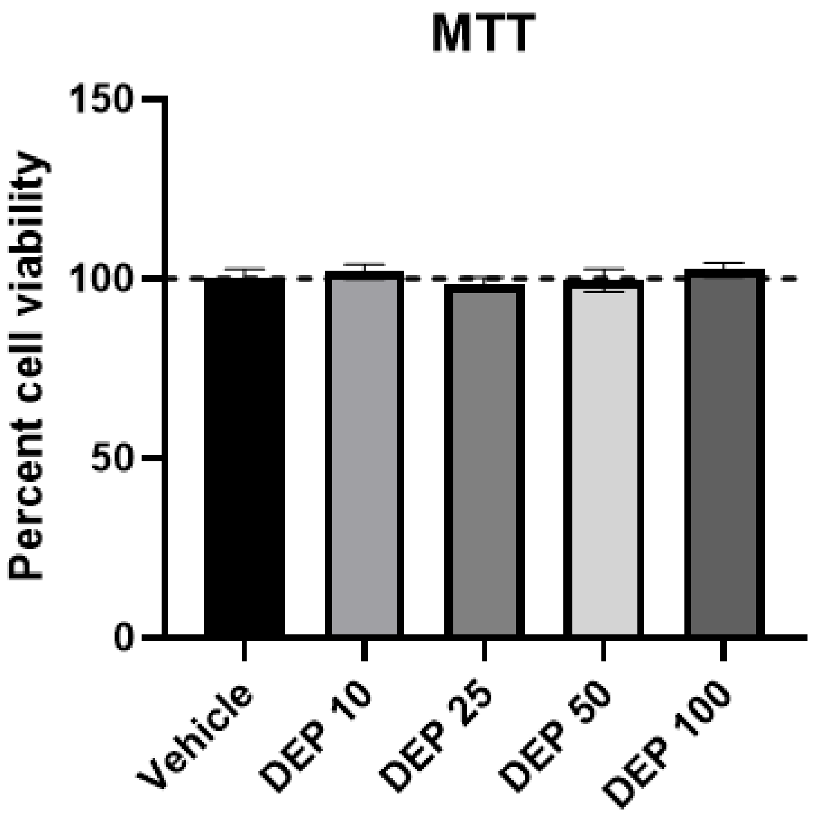

Supplementary Figure 5. HepG2 cells were treated with 10-100 µg/mL of DEP organic extract for 24 hours and percent cell viability was determined by an MTT assay relative to vehicle control. Values shown are mean ± SEM. Data was analyzed using one-way ANOVA followed by post-hoc Tukey’s test (n=10 replicates).

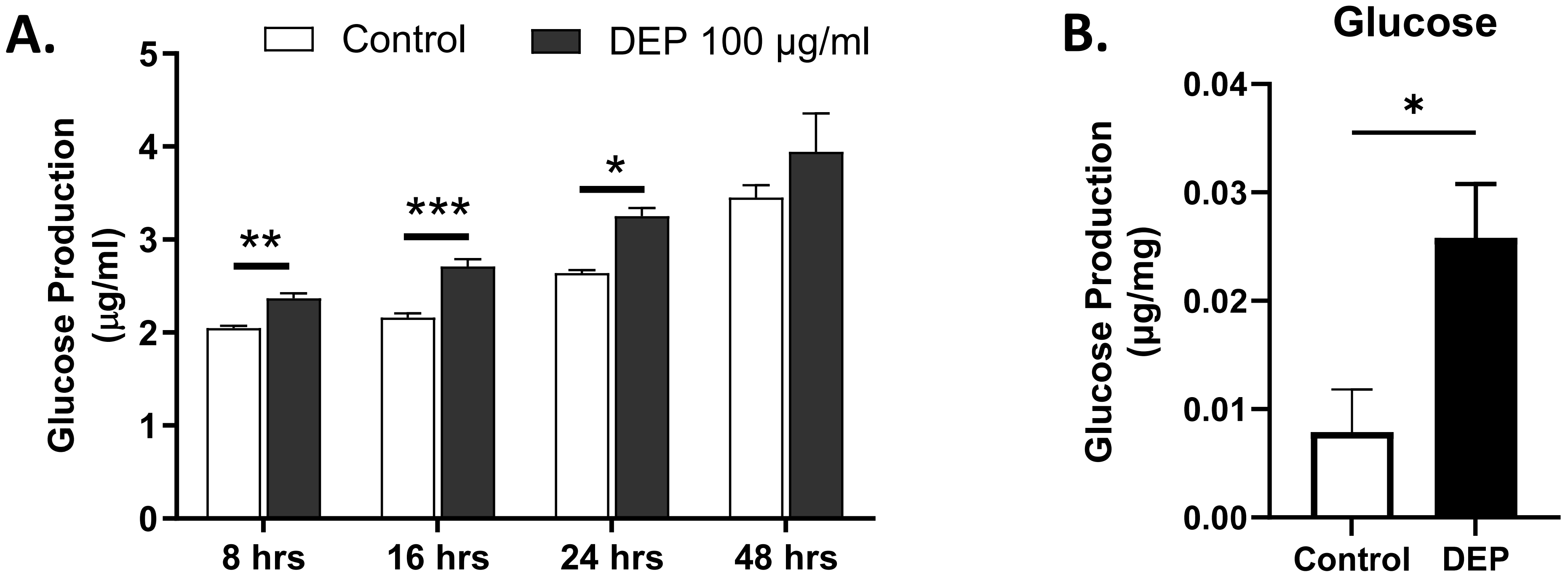

Supplementary Figure 6. (A) HepG2 cells were treated with 100 µg/mL of DEP organic extract vs. vehicle control for different durations (8, 16, 24 and 48 hours). **(B)** Glucose production with mouse primary hepatocytes treated with DEP extract at 25 µg/mL for 8 hours. Values shown are mean ± SEM. Data was analyzed using student's t-test. *p<0.05, **p<0.01 and ***p<0.001, DEP vs. vehicle control.

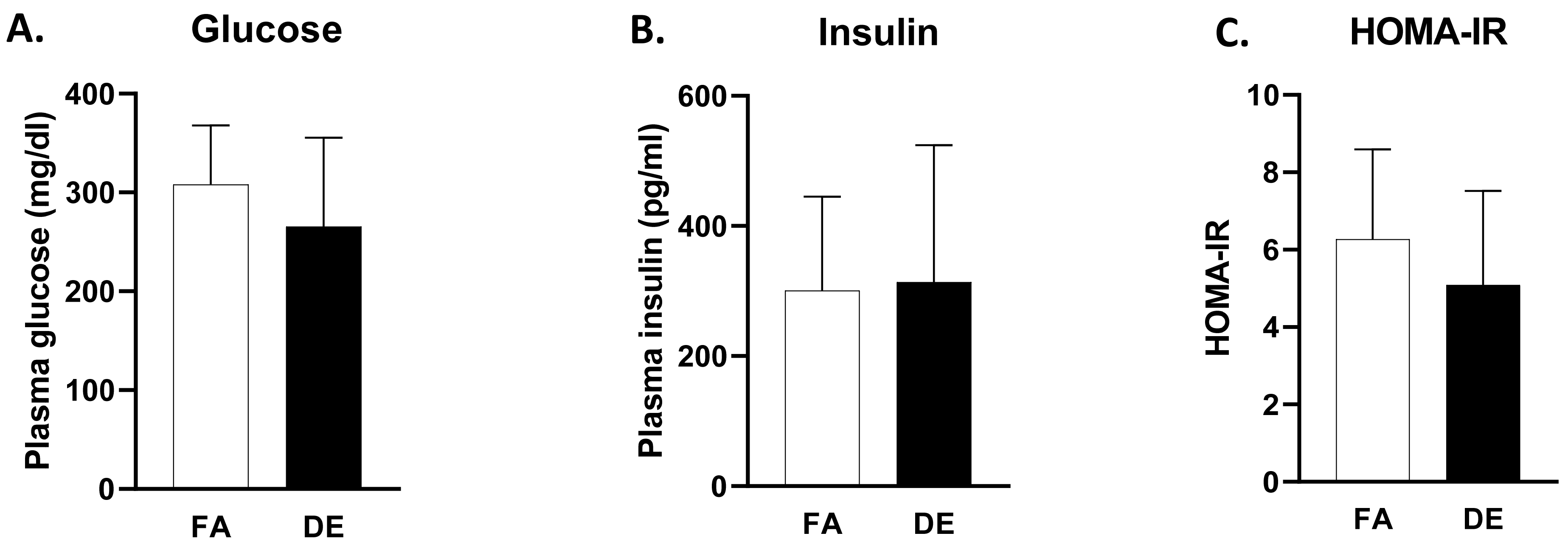

Supplementary Figure 7. Plasma levels of **(A)** glucose, **(B)** insulin and **(C)** HOMA-IR in FA (n=7) and DE (n=7) groups. Values shown are mean ± S.D.

Supplementary Table 1. Recovery of major organic fractions from 1 g of DEP

Fraction	Elution Solvent	Amount (mg)	Recovery (%)[a]
Aliphatic	Hexane	235	23.5
Aromatic	Hexane/methylene chloride (3/2, v/v)	105	10.5
Polar	Methylene chloride/methanol (1/1, v/v)	100	10
Total		440	44

[a]The amount of asphaltene from 1 g of DEP = 289.7 mg, which represents 29% of particle mass. This data has been published in Genome Biology (Gong et al, GGenome Biol. 2007;8 7:R149)

Supplementary Table 2. PAH content in each DEP fraction[a]

PAH	PAH Content (µg/g DEP)			
	Crude extract	Aliphatic	Aromatic	Polar
NAP	89	0.04	3.71	0
ACE	67	0	0.48	0
FLU	153	0	6.18	0
PHE	1576	0.04	110.87	0
ANT	24	0	0.95	0.12
FLT	678	0	45.13	1.01
PYR	530	0	23.37	0.05
BAA	91	0	6.18	0
CRY	158	0	7.7	0
BBF	48	0	1.71	0
BKF	20	0	0.29	0
BAP	16	0	0	0
DBA	16	0	0.1	0
BGP	18	0	0.1	0
IND	18	0	0.1	0

[a]PAH content was measured as previously described (40). Sixteen standard PAH were used to quantitate the PAH content in each fraction. NAP, naphthalene; ACE, acenaphthalene; FLU, fluorene; PHE, phenanthrene; ANT, anthracene; FLT, fluoranthene; PYR, pyrene; BAA, benzo(a)anthracene; CRY, chrysene; BBF, benzo(b)fluoranthene; BKF, benzo(k)fluoranthene; BAP, benzo(a)pyrene; DBA, dibenz(a,h)anthracene; BGP, benzo(ghi)perylene; IND, indeno(1,2,3,-cd)pyrene. This data has been published in Genome Biology (Gong et al, GGenome Biol. 2007;8 7:R149)

Supplementary Table 3. Quinone content in DEP fractions[a]

Quinones	Quinone Content in DEP Fractions (μg/g DEP)			
	Crude extract	Aliphatic	Aromatic	Polar
1,2 NQ	22.34	ND	ND	2.28
1,4 NQ	19.94	ND	ND	6.91
9,10 PQ	18.73	ND	ND	6.03
9, 10 AQ	69.34	ND	ND	36.86

[a]Quinone contents in crude DEP extract and fractions were analyzed as described in Materials and Methods. Four standard quinones were used for quantitation: 1,2 NQ, 1,2-naphthoquinone; 1,4 NQ, 1,4-naphthoquinone; 9,10 PQ, 9,10-phenanthrenequinone; and 9,10 AQ, 9,10-athraquinone. ND, not detected. This data has been published in Genome Biology (Gong et al, GGenome Biol. 2007;8 7:R149).

Supplementary Table 4. Primer sequences to amplify human candidate genes by qPCR.

Gene	Primers	
Pck1	Fwd 5' - 3'	agatggaggaagagggcatc
	Rev 5' - 3'	ggtcagtgagagccaacca
Igfbp1	Fwd 5' - 3'	aatggattttatcacagcagacag
	Rev 5' - 3'	ggtagacgcaccagcagagt
G6pc	Fwd 5' - 3'	agttgttgctggagtcctgtc
	Rev 5' - 3'	ggctggcattatagatgctgt
Foxo1	Fwd 5' - 3'	aagggtgacagcaacagctc
	Rev 5' - 3'	ttccttcattctgcacacga
B2M	Fwd 5' - 3'	ttctggcctggaggctatc
	Rev 5' - 3'	tcaggaaatttgactttccattc

Supplementary Table 5. Pathways affected by DE+FA exposure using MSigDB.

Gene Set Name	p-value	FDR q-value	Enrichment
METABOLISM OF LIPIDS AND LIPOPROTEINS	2.79E-12	5.30E-09	8.3
METABOLISM O AMINO ACIDS AND DERIVATIVES	5.47E-08	5.20E-05	10.4
FATTY ACID TRIACYLGLYCEROL AND KETONE BODY METABOLISM	1.42E-07	8.11E-05	11.1
CARBOXYLIC ACID METABOLIC_PROCESS	2.33E-07	8.11E-05	10.5
PPARA ACTIVATES GENE EXPRESSION	7.74E-07	1.84E-04	14.0
TRANSCRIPTION	9.16E-07	1.94E-04	4.4
AMINO SUGAR AND NUCLEOTIDE SUGAR METABOLISM	2.29E-06	3.11E-04	23.6
VALINE LEUCINE AND ISOLEUCINE DEGRADATION	2.29E-06	3.11E-04	23.6
TRANS GOLGI NETWORK VESICLE BUDDING	1.08E-05	1.05E-03	17.3
GLUCOSE METABOLISM	2.15E-05	1.83E-03	15.1
GLUCONEOGENESIS	2.16E-05	1.83E-03	24.5
METABOLISM OF CARBOHYDRATES	2.89E-05	2.20E-03	6.7
ABC TRANSPORTERS	6.08E-05	3.85E-03	18.9
GENERATION OF PRECURSOR METABOLITES AND ENERGY	3.35E-04	1.43E-02	8.5
PEROXISOME	5.62E-04	2.14E-02	10.7
